# A scoping review of perceptions from healthcare professionals on antipsychotic prescribing practices in acute care settings

**DOI:** 10.1186/s12913-022-08650-7

**Published:** 2022-10-21

**Authors:** Natalia Jaworska, Stephana J. Moss, Karla D. Krewulak, Zara Stelfox, Daniel J. Niven, Zahinoor Ismail, Lisa D. Burry, Kirsten M. Fiest

**Affiliations:** 1 Department of Critical Care Medicine, University of Calgary, Calgary, AB Canada; 2 Alberta Health Services, Calgary, AB Canada; 3 Faculty of Health, Dalhousie University, Halifax, NS Canada; 4 Department of Community Health Sciences, University of Calgary, Calgary, AB Canada; 5 O’Brien Institute for Public Health, University of Calgary, Calgary, AB Canada; 6 Department of Psychiatry, University of Calgary, Calgary, AB Canada; 7 Hotchkiss Brain Institute, University of Calgary, Calgary, AB Canada; 8 Departments of Pharmacy and Medicine, Leslie Dan Faculty of Pharmacy, Sinai Health System, University of Toronto, Toronto, Canada

**Keywords:** Antipsychotic medications, Prescribing practices, Deprescribing, Acute care, Critical care

## Abstract

**Background:**

Antipsychotic medications are frequently prescribed in acute care for clinical indications other than primary psychiatric disorders such as delirium. Unfortunately, they are commonly continued at hospital discharge and at follow-ups thereafter. The objective of this scoping review was to characterize antipsychotic medication prescribing practices, to describe healthcare professional perceptions on antipsychotic prescribing and deprescribing practices, and to report on antipsychotic deprescribing strategies within acute care.

**Methods:**

We searched MEDLINE, EMBASE, PsycINFO, CINAHL, and Web of Science databases from inception date to July 3, 2021 for published primary research studies reporting on antipsychotic medication prescribing and deprescribing practices, and perceptions on those practices within acute care. We included all study designs excluding protocols, editorials, opinion pieces, and systematic or scoping reviews. Two reviewers screened and abstracted data independently and in duplicate. The protocol was registered on Open Science Framework prior to data abstraction (10.17605/OSF.IO/W635Z).

**Results:**

Of 4528 studies screened, we included 80 studies. Healthcare professionals across all acute care settings (intensive care, inpatient, emergency department) perceived prescribing haloperidol (*n* = 36/36, 100%) most frequently, while measured prescribing practices reported common quetiapine prescribing (*n* = 26/36, 76%). Indications for antipsychotic prescribing were delirium (*n* = 48/69, 70%) and agitation (*n* = 20/69, 29%). Quetiapine (*n* = 18/18, 100%) was most frequently prescribed at hospital discharge. Three studies reported in-hospital antipsychotic deprescribing strategies focused on pharmacist-driven deprescribing authority, handoff tools, and educational sessions.

**Conclusions:**

Perceived antipsychotic prescribing practices differed from measured prescribing practices in acute care settings. Few in-hospital deprescribing strategies were described. Ongoing evaluation of antipsychotic deprescribing strategies are needed to evaluate their efficacy and risk.

**Supplementary Information:**

The online version contains supplementary material available at 10.1186/s12913-022-08650-7.

## Introduction

Antipsychotic medications, which are licensed for chronic psychiatric disease management, are frequently prescribed in hospital for acute clinical indications such as delirium [1–4]. These medications do not appear to alter the incidence or duration of delirium despite a large body of high-quality evidence evaluating their clinical efficacy [2, 5–7]. An increasing understanding of the potential risk of oversedation, falls, metabolic effects and cardiovascular morbidity related to antipsychotic medication use in acutely ill patients has translated into current guidelines recommending against the routine prescribing of antipsychotic medications in these clinical contexts [5, 8–16]. Antipsychotic medication prescribing for non-traditional indications in the acute care setting remains common and has been demonstrated to lead to antipsychotic prescription continuation at hospital discharge [17–20].

In-hospital deprescribing strategies defined as the deliberate and supervised reduction or withdrawal of an inappropriate or unnecessary medication may be a tool to reduce the proportion of patients being discharged from hospital with ongoing antipsychotic medications where the clinical indication may no longer be appropriate [21, 22]. However, in-hospital deprescribing strategies are infrequently implemented [23]. In-hospital clinical environments provide a safe and monitored setting to facilitate the necessary steps required to initiate a deprescribing care plan and warrants further evaluation.

Defining antipsychotic medication prescribing practices and the perceptions surrounding antipsychotic medication use in the acute care setting is essential to developing effective, sustainable, and collaborative multidisciplinary antipsychotic deprescribing strategies to promote appropriateness in prescribing and deprescribing during patient hospitalization [24–26]. The purpose of this scoping review is to synthesize the literature on antipsychotic medication prescribing practices in acute care, to describe healthcare professional perceptions on antipsychotic prescribing practices, and to report on antipsychotic deprescribing strategies within acute care.

## Methods

The scoping review research questions and methods for study selection and data charting were developed using the methodology described by Arksey and O’Malley and the Scoping Review Methods Manual proposed by the Joanna Briggs Institute [27, 28]. The scoping review protocol was registered (Open Science Framework:10.17605/OSF.IO/W635Z), and published as an open-access publication prior to data abstraction [29]. The review is reported according to the Preferred Reporting Items for Systematic Reviews and Meta-analysis Extension for Scoping Reviews (PRISMA-ScR) checklist [30]. Scoping review methodology was used to understand healthcare professional antipsychotic prescribing practices and perceptions to gain breadth and depth of understanding regarding this clinically relevant topic where previous comprehensive synthesis of the literature has not been completed.

This scoping review aims to answer two research questions:

1. What prescribing practices do healthcare professionals utilize to guide prescribing and deprescribing of newly initiated antipsychotic medications in patients prescribed an antipsychotic for clinical indications other than a primary psychiatric diagnosis in acute care?
2. What perceptions, facilitators and/or barriers do healthcare professionals identify that influence the way antipsychotic medications are prescribed or deprescribed in acute care for patients with a clinical indication other than a primary psychiatric diagnosis?

The components of population, exposure, comparator, outcome, study design, and timeframe were defined. The population included adult patients (as defined in the primary study) admitted to any acute care setting excluding care centres associating with the acute care setting (e.g., rehabilitation units), and healthcare professionals (e.g., physicians, nurses, pharmacists). The exposure was defined as antipsychotic medication administration for clinical indications other than a primary psychiatric diagnosis (e.g., psychosis, schizophrenia, bipolar disorder, major depressive disorder), dementia, or cognitive dysfunction (e.g., developmental disorders). Antipsychotic medications included in the search strategy were haloperidol/Haldol®, quetiapine/Seroquel® (immediate release and extended release), risperidone/Risperdal® (immediate release and extended release), ziprasidone/Zeldox®/Geodon®, aripiprazole/Abilify®, olanzapine/Zyprexa®, and methotrimeprazine/Nozinan®. This list of medications was selected as they form the most common clinically used antipsychotic medications in acute care based on clinical experience and from previous interventional and observational studies in the literature [18, 20, 31–33]. All comparators and comparisons were of interest. Outcomes of interest included antipsychotic medication prescribing practices (e.g., preferred antipsychotic prescribed, antipsychotic prescribed at hospital discharge, description of deprescribing initiatives) and perceptions of antipsychotic prescribing practices (e.g., perceptions on knowledge, prescribing capabilities and consequences) for all acute care patients excluding those with psychiatric diagnoses (e.g., psychosis, schizophrenia, bipolar disorder, major depressive disorder), dementia, or cognitive dysfunction (e.g., developmental disorders). Any observational or experimental and quasi-experimental original primary research study was included. Unpublished abstracts and studies of original research (i.e., conference abstracts) were included. Protocols, editorials, opinion pieces, systematic or scoping reviews were excluded. All publications from database inception to July 3, 2021 were considered.

### Data sources and searches

We systematically searched MEDLINE, EMBASE, PsycINFO, and CINAHL without restriction by date and language. Web of Science was searched for unpublished grey literature. The search strategy for MEDLINE was developed in consultation with a professional health sciences librarian (Supplementary Table 1). All database searches were performed on July 3, 2021 using search terms that included subject headings, keywords, and associated synonyms reflecting antipsychotic prescribing and deprescribing practices, and perceptions of all healthcare professionals on antipsychotic medication prescribing and deprescribing within acute care. Search terms included the following keywords: antipsychotic medications (as defined by the pre-specified medication list), prescribing practices, acute care setting, and perspectives. A pre-specified list of antipsychotic medications (Online Appendix 1) was selected for this scoping review to maintain a clinically relevant focus on the most common antipsychotics prescribed in acute care based on clinical experience and from previous observational and interventional studies on antipsychotic medication prescribing [31–35]. Reference lists of identified studies were additionally searched for relevant studies.

### Study selection

Studies were selected that reported on either antipsychotic prescribing and deprescribing practices or perceptions in acute care. We defined antipsychotic prescribing or deprescribing practices in acute care as perceived (i.e., participant reported) or measured prescribing or deprescribing practices in patients who did not have a psychiatric diagnosis, dementia, or cognitive dysfunction (e.g., developmental disorders) where chronic antipsychotic medication use may be clinically indicated. We included studies for adult patients (as defined in the primary study) that were hospitalized at or presenting to an acute care facility (e.g., critically ill, medical, surgical ward patients, or emergency department) and all healthcare professionals including, but not limited to physicians, nurses, and pharmacists. This study population was selected to reflect the population that is typically involved in the prescribing process of antipsychotic medications.

Studies identified through the bibliographic database search were first imported into Endnote-X9 (Clarivate, Philadelphia, USA) for de-duplication using the strategy outlined by Bramer et al*.* and subsequently imported for title/abstract and full-text review into Covidence (Veritas Health Innovation, Melbourne, Australia) [36]. Two reviewers (NJ, ZS) screened titles/abstracts and full-texts of studies independently and in duplicate for inclusion eligibility. Before each stage a calibration exercise was performed among reviewers to achieve > 75% interrater agreement in study selection. Titles and abstracts of studies identified from other sources (i.e., reference reviews, studies known to authors) were first screened for eligibility by the reviewers. Those studies meeting inclusion criteria subsequently underwent full-text review to determine inclusion for data extraction. Articles not available in English were translated using Google Translate, which has been reported as a reliable tool for translating documents for systematic reviews [37, 38]. Only those studies that satisfied all inclusion criteria were selected for data extraction. Conference abstracts were included if they met all inclusion criteria even if full articles related to the conference abstracts were not found. Full texts related to included conference abstracts were searched for and if full texts were available, full texts were selected for inclusion over the conference abstract to avoid duplication. Two reviewer agreement was required for studies to proceed on to data extraction. Disagreements regarding study selection were resolved through discussion between the reviewers.

### Data extraction

Two reviewers (NJ, SJM) completed a calibration exercise on ten studies to achieve > 75% interrater agreement prior to data extraction. Data were extracted by two reviewers (NJ, SJM) independently and in duplicate using a standardized data extraction form. We extracted the following data: study identifiers and type (e.g., study location, study design, sample size, study setting), participants (e.g., healthcare professionals, patients), exposure (e.g., antipsychotic type, antipsychotic dosing), and outcome (e.g., perceived or measured antipsychotic prescribing practices, antipsychotic medication prescribed at hospital discharge, antipsychotic knowledge and perceptions) as well as information on antipsychotic deprescribing approaches and strategies. We contacted corresponding authors via email with no follow-up email for clarification if no specific antipsychotic medication was defined in the study.

### Data synthesis and analysis

Studies were summarized following validated guidelines for narrative synthesis of quantitative studies [30, 39]. Considering heterogeneous quantitative data from included studies, we grouped studies according to outcomes and setting (i.e., intensive care, inpatient, emergency department) and summarized data as counts with proportions.

Studies describing perceptions of healthcare professionals on antipsychotic medication prescribing were evaluated by deductive qualitative thematic analysis utilizing the Theoretical Domains Framework (TDF). The TDF is a theoretical framework of 14 behavior and behavior change domains with associated constructs that identifies pertinent factors that influence the behavior patterns of healthcare professionals [39, 40]. Qualitative thematic analysis was performed to understand the reported priority factors that influence healthcare professional antipsychotic prescribing practices. We used a two-stage approach described by Braun & Clarke to evaluate included studies [41]. One reviewer (NJ) completed analysis for all included studies with second reviewer (SJM) verifying the data for accuracy. In the first stage, text from included studies was read line-by-line to identify and categorize specific codes to the TDF domains [41]. In the second stage, text was analyzed for discrete TDF constructs within each domain [39]. Disagreements in coding of text to a domain or construct were resolved through discussion between the two reviewers. All studies for qualitative analysis were in English and did not require translation.

Our methodology aligned with our previously published protocol. As there were no studies identified that reported on the perceptions of patients and their families regarding antipsychotic prescribing practices, we were unable to report on these outcomes that were outlined in our published protocol [29].

## Results

**Fig. 1:**
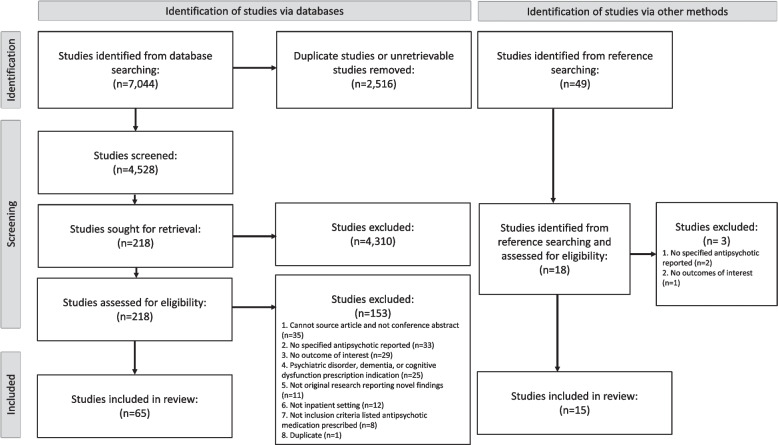
Study selection flow chart

We identified 4,528 unique studies, of which 218 full texts were reviewed and 65 studies were included. An additional 49 studies were identified from reference searching, of which an additional 15 studies were included totalling 80 eligible studies (Online Appendix 2). Fifteen studies were included in the form of conference abstracts. Most studies were excluded as they did not report on a specific antipsychotic medication (*n* = 33/153, 22%) (Fig. 1).

### Description of studies

Studies were conducted between 1996 to 2021 (inclusive) with most studies being carried out between 2016 to 2018 (Supplementary Fig. 1). Most studies were conducted in North America (*n* = 42/80, 53%), Europe (*n* = 16/80, 20%), or Asia (*n* = 8/80, 10%) and evaluated the intensive care (*n* = 49/80, 61%), inpatient non-intensive care setting (*n* = 27/80, 34%) or emergency department setting (*n* = 5/80, 6%) (Supplementary Fig. 2). One study reported on both the intensive care and inpatient setting. Studies included healthcare professionals (*n* = 36/80, 45%), patients (*n* = 42/80, 53%), or both healthcare professionals and patients (*n* = 2/80, 3%). All studies describing perceptions on antipsychotic medication prescribing were comprised of healthcare professionals, namely physicians (including physician assistants and nurse practitioners), nurses, pharmacists, and respiratory therapists. Study characteristics are listed in Supplementary Table 2.

### Antipsychotic prescribing practices across acute care settings

Of the included studies most studies described either participant-reported prescribing practices (intensive care, *n* = 24/49, 49%; inpatient, *n* = 8/27, 30%; emergency department, *n* = 4/5, 80%), measured (i.e., actual) prescribing practices (intensive care, *n* = 16/49, 33%; inpatient, *n* = 14/27, 52%), or characterized the monitoring and management of pain, agitation, or delirium (intensive care, *n* = 19/49, 39%; inpatient, *n* = 1/27, 4%) (Table 1; Supplementary Table 3).

**Table 1:** Measured or reported outcomes evaluated on antipsychotic medication prescribing practices in included studies, by acute care setting

MEASURED OR REPORTED OUTCOMES	ACUTE CARE SETTING (Number of studies)		
	**Intensive care**^**a **^(*N* = 49)	**Inpatient**^**a **^(*N* = 27)	**Emergency department** (*N* = 5)
Participant reported prescribing practices	24 (49%)	8 (30%)	4 (80%)
Measured prescribing practices	16 (33%)	14 (52%)	0 (0%)
Characterize monitoring and management of pain, agitation, or delirium	19 (39%)	1 (4%)	0 (0%)
Measured prescribing practices at transitions of care	9 (18%)	3 (11%)	0 (0%)
Antipsychotic deprescribing	3 (6%)	0 (0%)	0 (0%)
Evaluation of Inappropriate antipsychotic prescribing practices	1 (2%)	2 (7%)	0 (0%)
Delirium outcomes^b^	2 (4%)	1 (4%)	0 (0%)
Mortality	2 (4%)	1 (4%)	0 (0%)
Sedation effects	1 (2%)	1 (4%)	1 (20%)
Prescribing practice audit	1 (2%)	0 (0%)	0 (0%)
Falls	0 (0%)	1 (4%)	0 (0%)

Percentages do not add up to 100 due to the possibility of multiple outcomes per study

^a^One primary study reports combined outcomes for patients admitted as inpatients and in ICU and results reported in both categories

^b^Includes days-free of delirium and delirium resolution

Most studies (*n* = 69/80, 86%) described the clinical indications for antipsychotic prescribing. Delirium (intensive care, *n* = 34/43, 79%; inpatient, *n* = 14/21, 67%) and agitation (intensive care, *n* = 9/43, 21%; inpatient, *n* = 7/21, 33%) were the most common clinical indications for antipsychotic prescribing in the intensive care and inpatient setting. Agitation was the most common clinical indication for antipsychotic prescribing in the emergency department (*n* = 4/5, 75%) (Supplementary Tables 4 and 5).

**Fig. 2:**
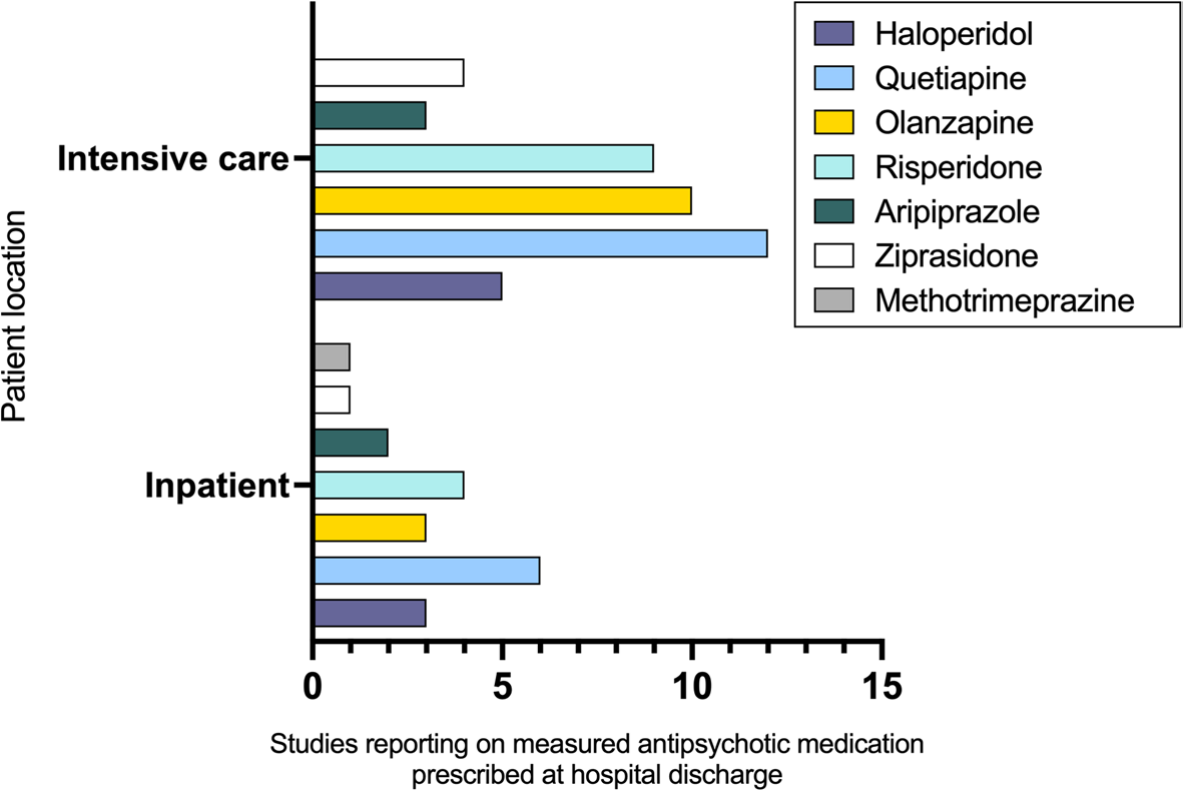
Measured antipsychotic medications prescribed at hospital discharge for included studies on antipsychotic prescribing practices, by acute care setting^1^. Patient location defined as the primary location patients were first admitted and started on antipsychotic medications. Patients admitted to intensive care were either discharged direclty home or to the hospital ward before hospital discharge. Patients admitted to inpatient setting were never admitted to intensive care. ^1^No studies reporting measured outcomes included patients from the emergency department setting

In all three settings, haloperidol was perceived to be the most common prescribed antipsychotic medication in studies describing healthcare professional-reported antipsychotic prescribing practices (intensive care, *n* = 24/24, 100%; inpatient, *n* = 9/10, 90%; emergency department, *n* = 4/4, 100%) (Supplementary Tables 5 and 6). Evaluation of measured (i.e., actual) antipsychotic prescribing practices identified 34 studies (intensive care, *n* = 20/34, 59%; inpatient *n* = 14/34, 42%; emergency department *n* = 0/34, 0%). In both the intensive care and inpatient setting, haloperidol remained a common prescribed antipsychotic medication (intensive care, *n* = 14/20, 70%; inpatient, *n* = 12/14, 86%). However, quetiapine (intensive care, *n* = 17/20, 85%; inpatient, *n* = 9/14, 85%), olanzapine (intensive care, *n* = 14/20, 70%; inpatient, *n* = 9/14, 70%), and risperidone (intensive care, *n* = 13/22, 65%; inpatient, *n* = 9/14, 65%) were additionally commonly measured as being prescribed in these settings (Supplementary Tables 5 and 7). Figure 2 demonstrates the results of studies reporting on measured antipsychotic medications prescribed at hospital discharge by setting and antipsychotic medication type. In both the intensive care and inpatient setting, quetiapine was reported in all studies to be most often antipsychotic continued at hospital discharge (intensive care, *n* = 12/12, 100%; inpatient, *n* = 6/6, 100%). No studies were identified reporting on antipsychotic prescribing at hospital discharge in the emergency room setting.

Co-prescription of sedative hypnotic medications in addition to antipsychotic medications occurred in the intensive care and inpatient setting. Benzodiazepines (intensive care, *n* = 24/28, 86%; inpatient, *n* = 8/15, 53%), intravenous sedation infusions such as propofol or ketamine infusions (intensive care, *n* = 9/28, 32%), and other additional antipsychotics (inpatient, *n* = 8/15, 53%) were most commonly reported to be co-prescribed with antipsychotic medications (Supplementary Tables 8 and 9).

### Perceptions on antipsychotic prescribing practices

**Fig. 3:**
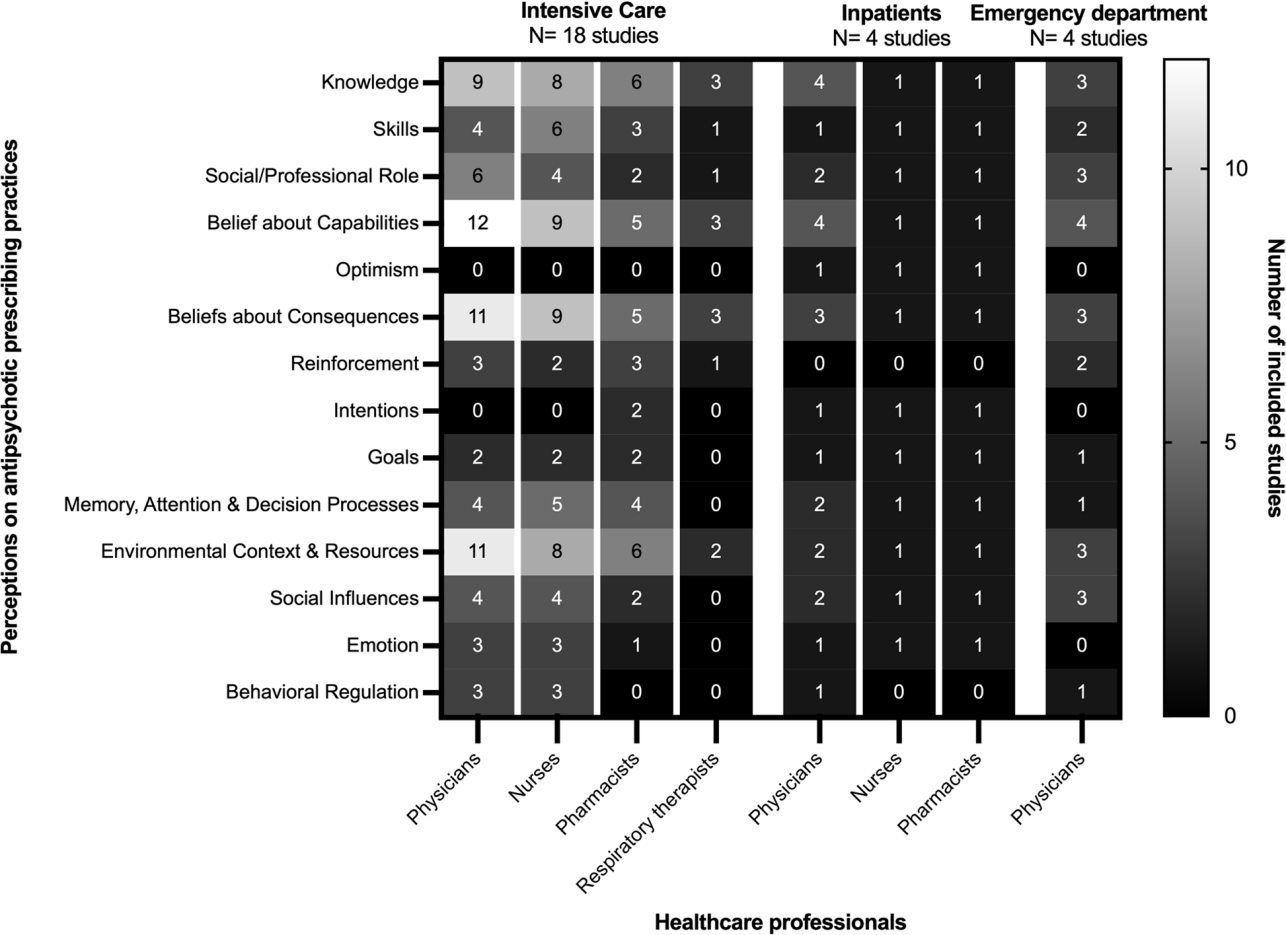
Total number of studies^1^ reporting on perceptions toward antipsychotic prescribing practices according to the Theoretical Domains Framework, by acute care setting and healthcare professional role. ^1^Four unique studies not included due to no reported health care professional role

Figure 3 delineates the perspectives of healthcare professionals on antipsychotic medication prescribing practices from 29 included studies (*n* = 29/80, 36%) organized according to the domains of the TDF and by setting. Most included studies describe perspectives in the intensive care setting (*n* = 18/29, 62%). Perspectives across all three settings were related to knowledge (e.g., knowledge of conditions requiring antipsychotics) (*n* = 23/29, 79%), beliefs about capabilities (e.g., perceived competence regarding antipsychotic prescribing contexts such as delirium) (*n* = 25/29, 86%), beliefs about consequences (e.g., beliefs surrounding antipsychotic efficacy for delirium) (*n* = 23/29, 79%), and environmental context and resources (e.g., screening tools and protocols to guide antipsychotic prescribing) (*n* = 21/29, 72%) (Supplementary Table 10). Additional thematic analysis delineating TDF domains and constructs by each included study is available in Supplementary Table 11.

Healthcare professionals reported feeling knowledgeable about the diagnosis of delirium as well as the use and efficacy of antipsychotics to treat delirium. However, some healthcare professionals reported having low in-depth knowledge of guideline recommendations on the use of antipsychotics in the setting of delirium. Overall, healthcare professionals were confident in their capabilities to prescribe antipsychotic medications but stated that this was largely from clinical experience rather than through formal training. Healthcare professionals perceived antipsychotics as an effective adjunct for the management of delirium that did not pose a high risk of adverse events to limit their prescribing practices. Healthcare professionals reported concern regarding the consequences of delirium that was not treated with antipsychotics. Environmental factors including the use of delirium screening tools to detect delirium were reported to influence healthcare professional antipsychotic prescribing practices. The lack of antipsychotic prescribing protocols to support these screening tools were cited as influencing how and when antipsychotics were prescribed.

### In-hospital antipsychotic deprescribing strategies

Three (*n* = 3/80, 4%) studies described antipsychotic medication deprescribing strategies in the acute care settings (Supplementary Table 12). Two studies described a pharmacist-based intervention either in the form of an electronic handoff tool or the use of prescriptive authority to deprescribe antipsychotic medications once the acute clinical indication had resolved. One study described the use of an antipsychotic discontinuation algorithm implemented prior to transfer out of the intensive care unit. Two of the studies additionally described the use of education (pharmacist and multidisciplinary) regarding consensus guidelines on antipsychotic medication use in intensive care.

## Discussion

We synthesized the evidence evaluating antipsychotic prescribing practices and the perceptions of healthcare professionals that influence the way this class of medications are prescribed for non-psychiatric diagnoses in acute care. Delirium and agitation are reported as the most frequent indications for antipsychotic prescribing. Across all acute care settings haloperidol was perceived as the most frequently utilized antipsychotic. In contrast, within the intensive care and inpatient care settings actual antipsychotic prescribing practices identified prevalent use of atypical antipsychotics with quetiapine being the most frequently prescribed antipsychotic medication. Perceived antipsychotic prescribing practices differed from actual measured antipsychotic prescribing practices and may impact how antipsychotic medications are prescribed at hospital discharge. In both the intensive care and inpatient settings, we found that quetiapine was the most frequently prescribed antipsychotic medication at hospital discharge and more accurately reflected measured actual antipsychotic prescribing practices than perceived antipsychotic prescribing practices.

Our findings identifying differing perceived versus actual measured antipsychotic prescribing practices has not previously been described. An explanation for these differences was not identified in our scoping review. Further evaluation using qualitative interviews with healthcare professionals to explore the cause of this discrepancy in-depth are needed. It is possible that established high-quality evidence describing the increased risk of cardiovascular mortality and neurologic complications related to haloperidol use may be playing a role in the decreased measured utilization of haloperidol despite reported preferences for haloperidol [42–44]. Despite a growing body of evidence focused on the clinical efficacy of antipsychotic medication use in delirium in both in the inpatient and intensive care setting demonstrating limited efficacy in mediating the prevention or duration of delirium, healthcare professionals continue to report prescribing antipsychotic medications [5–7, 45]. The prevalent use of quetiapine and its ongoing prescription at hospital discharge may reflect a new repurposing of this antipsychotic for sleep management following the resolution of delirium or agitation given its histaminergic properties [46]. Further, in the intensive care setting limited alternative sedation-sparing medications available for the management of the symptoms of agitation or delirium likely remains a common driver of quetiapine prescriptions [2, 7, 8].

Our study expands on healthcare professional prescribing perceptions specific to antipsychotic medications. Healthcare professionals felt confident in their antipsychotic prescribing abilities and identified antipsychotics as an effective clinical tool that do not carry sufficient risk of adverse events to limit their prescribing. Further, environmental factors such as delirium screening tools and the lack of established antipsychotic prescribing protocols to support these screening tools appear to influence healthcare professional prescribing practices. Few current studies are available that address in-hospital deprescribing strategies to reduce ongoing antipsychotic medication prescribing at hospital discharge [47–49]. Studies reporting on deprescribing strategies have been limited to the intensive care setting focusing on education initiatives and algorithmic deprescribing pathways with variable efficacy in sustainably reducing antipsychotic medication prescribing at hospital discharge [47, 48]. The implications of these results suggest that an approach addressing individual prescribing practice beliefs as well as targeting established health system processes through protocolized pathways that provide stepwise escalation and de-escalation recommendations for antipsychotic dosing for healthcare professionals may be effective in establishing sustainable reductions in antipsychotic medication prescriptions continued at hospital discharge [50]. Further, formalized training and education for healthcare professionals on their antipsychotic medication prescribing practices may be an additional effective tool that should be prioritized to ensure healthcare professionals are aware of the discrepancy between their perceived and measured prescribing practices.

Our study has multiple strengths and notable limitations. We utilized a broad and comprehensive search strategy of multiple databases without restrictions including a grey literature search. Despite a comprehensive and exhaustive search strategy of the literature, it is possible that some relevant studies may have been missed as we limited our list of antipsychotic medications selected for this scoping review to focus on the most clinically relevant antipsychotic medications prescribed within acute care identified in the current literature and known to be utilized from clinical experience [32–35]. Limiting the search strategy to this antipsychotic medication list aimed to ensure feasibility, minimize heterogeneity of the data, and emphasize clinical applicability. However, generalizability may be limited in clinical environments where other antipsychotic medications may be more frequently used (e.g., low health resource clinical environments) and the results may be applicable to only certain countries (e.g., specific antipsychotics approved for use in the country). Lastly, few studies were identified regarding the antipsychotic prescribing practices within the emergency department and limited conclusions can be drawn regarding antipsychotic prescribing practices in this clinical setting.

## Conclusions

Perceived antipsychotic prescribing practices differ from actual measured antipsychotic prescribing practices in acute care with more frequent prescribing of atypical antipsychotic medications in-hospital and at hospital discharge. Deprescribing strategies were infrequently described in the literature. Further research is needed to understand the reasons for inconsistencies between perceived and actual antipsychotic prescribing to develop and evaluate in-hospital antipsychotic deprescribing strategies.

## Supplementary Information

**Additional file 1.** 

**Additional file 2.** 

**Additional file 3: Supplementary Table 1.** Search strategy used in MEDLINE. **Supplementary Table 2.** Characteristics of included studies. **Supplementary Table 3.** Antipsychotic reported outcomes of included studies. **Supplementary Table 4.** Reportedantipsychotic medication prescribing indications included studies by acute care setting. **Supplementary Table 5.** Measured and perceived antipsychotics prescribed and prescribing indications reported for included studies, by acutecare setting. **Supplementary Table 6.** Number of studies reporting on healthcare professional reported perceived antipsychotic prescribing practices in acute care, by acute care setting and antipsychotic type. **Supplementary Table 7.** Number of studies reporting on measured outcomes of antipsychotic prescribing practices in the acute care setting, by acute care setting and antipsychotic type. **Supplementary Table 8.** Reported additionally prescribed sedative hypnotic medications for included studies reporting on antipsychotic medication prescribing, by acute care setting. **Supplementary Table 9.** Reported co-prescribed sedative hypnotic medications with antipsychotic medications for included studies which report on additionally prescribed medications, by acute care setting. **Supplementary Table 10. **Domains and constructs according to the Theoretical Domains Framework of perspectives on antipsychotic prescribing from healthcare professionals for included studies, by acute care setting. **Supplementary Table 11.** Deductive thematic analysis using the Theoretical Domains Framework on perceptions on antipsychotic prescribing forincluded studies. **Supplementary Table 12.** Description of reported antipsychotic deprescribing strategies applied in parallel for included studies reporting on antipsychotic medication prescribing.

## Data Availability

All data generated or analyzed during this study are included in this published article and its supplementary information files.

## Abbreviation

TDF: Theoretical Domains Framework
