# Supplementary material for: A scoping review of perceptions from healthcare professionals on antipsychotic prescribing practices in acute care settings
Source: BMC Health Serv Res. 2022 Oct 21;22:1272. doi: 10.1186/s12913-022-08650-7 (PMC9587627; doi:10.1186/s12913-022-08650-7)
Supplement: Supplementary file 1 — Additional file 1. [file 12913_2022_8650_MOESM1_ESM.docx]

**Perceptions from patients, family members, and healthcare professionals in acute care settings on antipsychotic prescribing practices: A scoping review protocol**

**Registration:**

This protocol provides the methods that we will use in the scoping review. The scoping review protocol was developed following the methodology from the Scoping Review Methods Manual proposed by the Joanna Briggs Institute.^1^ We will follow the Preferred Reporting Items for Systematic Review and Meta-Analyses Protocols (PRISMA-P) guideline for reporting, and we will register the protocol on the Open Science Framework website prior to data extraction and analysis.^2^

**Authors:**

1. Natalia Jaworska MD MSc, Department of Critical Care Medicine, Alberta Health Services & University of Calgary, Calgary, AB, Canada; njaworsk@ucalgary.ca
   1. Author contributions: Conception and development of research question, research design development, acquisition of data, interpretation of data, manuscript formation, managing submission process.
2. Stephana J. Moss MSc, Department of Critical Care Medicine, University of Calgary, Calgary, AB, Canada; stephana.moss@ucalgary.ca
   1. Author contributions: Conception and development of research question, research design development, acquisition of data, interpretation of data, manuscript formation, managing submission process.
3. Zara Stelfox, Department of Critical Care Medicine, University of Calgary, Calgary, AB, Canada; zstelfox@gmail.ca
   1. Author contributions: Conception and development of research question, research design development, acquisition of data, interpretation of data, manuscript formation, managing submission process.
4. Karla D. Krewulak PhD, Department of Critical Care Medicine, Alberta Health Services & University of Calgary, Calgary, AB, Canada; kkrewula@ucalgary.ca
   1. Author contributions: Conception and development of research question, research design development, acquisition of data, interpretation of data, manuscript formation.
5. Kirsten M. Fiest PhD, Department of Community Health Sciences & O’Brien Institute for Public Health, Department of Psychiatry & Hotchkiss Brain Institute, Department of Critical Care Medicine, Alberta Health Services & University of Calgary, Calgary, AB, Canada; kmfiest@ucalgary.ca
   1. Author contributions: Conception and development of research question, research design development, acquisition of data, interpretation of data, manuscript formation.

**Rationale:**

Antipsychotic medications are frequently prescribed to hospitalized patients for off-label clinical indications.^3^ These medications are also commonly continued at the time of hospital discharge.^4^ Among critically ill adult patients, up to 30% of patients prescribed an antipsychotic medication in the ICU will subsequently be discharged from hospital with an ongoing prescription without clinical indication.^4,5^

Long-term antipsychotic medication use is associated with increased risk of sudden cardiac death, falls, and worsening cognitive impairment.^6,7^ Characterizing prescribing and deprescribing practices of antipsychotic medications in hospital is important to understand the facilitators and barriers to effective, sustainable antipsychotic deprescribing strategies.^7^ A knowledge gap exists in the understanding of the factors that influence antipsychotic medication prescribing practices among healthcare providers.

Defining the current scope of literature of the perceptions, experiences, facilitators and/or barriers of healthcare providers, patients, families is important to understanding the facilitators and barriers to deprescribing antipsychotic medications. Collaborative multidisciplinary efforts to modify prescribing practices of antipsychotics are needed to promote rational prescribing and deprescribing of these medications during hospitalization.^8,9^ A scoping review will be completed to identify, describe, and characterize antipsychotic medication prescribing practices and their impact on patients, families, and healthcare providers. The results of this scoping review will help inform future interventional strategies to facilitate in-hospital antipsychotic medication deprescribing for hospitalized patients and improve patient safety through responsible antipsychotic prescribing practices.

**Review Objectives:**

### *Objective*

To synthesize the literature reporting on antipsychotic medication prescribing practices in acute care settings, describe perspectives on prescribing practices among patients, families, and healthcare professionals, and identify facilitators and/or barriers aimed at improving prescribing practices.

### *Aim*

To identify knowledge gaps and patterns in existing literature reporting on antipsychotic medication prescribing practices in acute care settings that could be adapted or adopted through quality improvement initiatives and tested in future research.

**Methods**

***Eligibility criteria***

See Table 1 for all inclusion and exclusion criteria. The components of population, exposure, comparator, outcome, study design, and timeframe will be defined as follows:

## Population, clinical indication(s), and condition(s): Adult patients greater than 18 years of age admitted to any acute care setting excluding care centres associating with the acute care setting, their family members, and healthcare professionals

## Interventions/Exposure: Antipsychotic medication administration for clinical indications other than a primary psychiatric diagnosis (ie. psychosis, schizophrenia, bipolar disorder). Antipsychotic medications will include: haloperidol/Haldol®, quetiapine/Seroquel®, risperidone/Risperidal®, ziprasidone/Zeldox®/Geodon®, aripiprazole/Abilify®, olanzapine/Zyprexa®, methotrimeprazine/Nozinan®

## Comparator(s): All comparators and comparisons are of interest

### Outcomes: Perceptions of antipsychotic prescribing practices, which may include facilitators and/or barriers. Antipsychotic medication prescribing practices/patterns, which may include prescribing and deprescribing will also be included.

- Study Design: Any observational or experimental and quasi-experimental primary peer-reviewed research study including randomized control trials, non-randomized control trials, interrupted time-series, before and after studies, semi-structured interviews, focus groups, qualitative studies, surveys, case studies and case reports. Protocols, editorial, opinion piece, systematic or scoping review will be excluded.
- Timeframe: No time limit on publication date will be set

***Information sources***

MEDLINE, EMBASE, PsycINFO, and CINAHL will be searched for key words relating to antipsychotic medication prescribing practices and experiences. Web of Science will be searched for unpublished grey literature. All databases will be searched on the same day in July 2021. No date restrictions will be included.

***Search strategy***

We will follow the Preferred Reporting Items for Systematic Review and Meta-Analyses Extension for Scoping Reviews (PRISMA-ScR) checklist for reporting.^10^ The search strategy for MEDLINE has been developed in consultation with a professional health services librarian. The search strategy for the other databases have been adapted from the MEDLINE strategy. A broad range of search terms that include subject headings and keywords have been selected that reflect antipsychotic medications prescribing practices and perspectives of healthcare providers on antipsychotic medication prescribing and deprescribing in hospitalized patients. Search terms are focused on adult critically ill and hospitalized patients, family members, and healthcare providers, antipsychotic medication administration, prescribing and deprescribing practices (including facilitators, barriers, or strategies), and patient or provider perspectives, and experiences. (see Appendix 1 for full search strategy). Studies published in any language will be considered. There are no limitations to the date of publication.

*Study records*

Records identified through the search will be managed using Endnote-X9 (Clarivate, Philadelphia, USA). The primary reviewer (NJ) will remove duplicates using the de-duplication strategy outlined by Bramer *et al* prior to importing titles and abstracts for review into Covidence (Veritas Health Innovation, Melbourne, Australia).^11^

*Selection process*

Figure 1 includes the exclusion flow chart for all screening steps. Prior to the screening of titles and abstracts, two reviewers (NJ, ZS) will complete a calibration exercise of at least 50 random citations to ensure 100% agreement before commencing the full titles and abstracts screening protocol for eligible studies. The same two reviewers (NJ, ZS) will then independently and in duplicate complete screening of titles and abstracts for the selection of eligible studies for potential inclusion. Any study selected at this stage by any of the reviewers will proceed on to the next stage. After initial screening, one reviewer (ZS) will retrieve all articles identified for full-text screening. Two reviewers (NJ, SM) will then review full-texts independently and in duplicate for inclusion eligibility and for the development of the data abstraction table. We will complete an additional calibration exercise for the screening of reference lists of articles meeting eligibility criteria. The same two reviewers (NJ, SM) will screen reference lists independently and in duplicate for potentially relevant articles. If articles are not available in English, they will be translated either through a reviewer with fluency in the specific language or using Google Translate, which has been identified to be reliable for translating documents for systematic reviews.^12^ Disagreements in study selection will again be resolved by a third reviewer through discussion and if necessary, with a third reviewer (KK).

*Data collection process*

A data abstraction form will be created in Microsoft Excel with an associated data dictionary of allowable variables. Data abstraction will include study identifiers, study design, included participant numbers and demographics (role, age, sex/gender, ethnicity), antipsychotic medication exposure type, and outcome information with prescribing or deprescribing strategies if applicable. We will pilot the data abstraction form with two reviewers with a subset of studies to ensure clarity and consistent abstraction. We will summarize perspectives, experiences, facilitators, or barriers qualitatively using the Theoretical Domains Framework (TDF) for analysis described further below. Two reviewers (NJ, SM) will independently complete data abstraction with the first reviewer completing data abstraction for all included studies and the second reviewer comparing extracted data for accuracy. Disagreements will be resolved through discussion or by a third reviewer (KK), if necessary. For any missing or unclear data that cannot be extracted, we will contact the corresponding author via email for clarification. A four-week period will be allocated for responses with one follow-up email planned at the two-week interval within that period if no response is received.

*Risk of bias*

The methodological quality of included studies will not be assessed as the aim of this scoping review is to identify potential prescribing and deprescribing practices and strategies, as well as stakeholder perspectives, facilitators, and barriers to antipsychotic medication deprescribing.

**References**

1. Peters MDJ GC, McInerney P, Munn Z, Tricco AC, Khalil, H. Chapter 11: Scoping Reviews (2020 version). In: In: Aromataris E MZE, editor. JBI Manual for Evidence Synthesis, JBI; 2020.

2. Moher D, Shamseer L, Clarke M, et al. Preferred reporting items for systematic review and meta-analysis protocols (PRISMA-P) 2015 statement. *Systematic Reviews* 2015; **4**(1): 1.

3. Maher AR, Maglione M, Bagley S, et al. Efficacy and Comparative Effectiveness of Atypical Antipsychotic Medications for Off-Label Uses in Adults: A Systematic Review and Meta-analysis. *JAMA* 2011; **306**(12): 1359-69.

4. Tomichek JE, Stollings JL, Pandharipande PP, Chandrasekhar R, Ely EW, Girard TD. Antipsychotic prescribing patterns during and after critical illness: a prospective cohort study. *Crit Care* 2016; **20**(1): 378.

5. Kram BL, Kram SJ, Brooks KR. Implications of atypical antipsychotic prescribing in the intensive care unit. *J Crit Care* 2015; **30**(4): 814-8.

6. Pandharipande PP, Girard TD, Jackson JC, et al. Long-term cognitive impairment after critical illness. *N Engl J Med* 2013; **369**(14): 1306-16.

7. Girard TD. Sedation, Delirium, and Cognitive Function After Critical Illness. *Crit Care Clin* 2018; **34**(4): 585-98.

8. Devlin JW, Smithburger P, Kane JM, Fraser GL, Skrobik Y. Intended and Unintended Consequences of Constraining Clinician Prescribing: The Case of Antipsychotics. *Crit Care Med* 2016; **44**(10): 1805-7.

9. Young SL, Taylor M, Lawrie SM. "First do no harm." A systematic review of the prevalence and management of antipsychotic adverse effects. *J Psychopharmacol* 2015; **29**(4): 353-62.

10. Tricco AC, Lillie E, Zarin W, et al. PRISMA Extension for Scoping Reviews (PRISMA-ScR): Checklist and Explanation. *Ann Intern Med* 2018; **169**(7): 467-73.

11. Bramer WM, Giustini D, de Jonge GB, Holland L, Bekhuis T. De-duplication of database search results for systematic reviews in EndNote. *J Med Libr Assoc* 2016; **104**(3): 240-3.

12. Jackson JL, Kuriyama A, Anton A, et al. The Accuracy of Google Translate for Abstracting Data From Non–English-Language Trials for Systematic Reviews. *Annals of Internal Medicine* 2019; **171**(9): 677-9.

**Table 1.** Inclusion and exclusion criteria

| **Inclusion** | **Exclusion** |
| --- | --- |
| Any observational, experimental, or quasi-experimental original peer-reviewed research study | The study is a study protocol, editorial, opinion piece, systematic or scoping review, or has not been peer-reviewed |
| The study population includes patients, their family members and/or healthcare providers of hospitalized or critical care adult patients | The study population includes patients, family members and/or healthcare providers of hospitalized children, or neonates  The study population includes patients, family members, or healthcare providers of those being treated for a primary psychiatric disorder |
| The study describes antipsychotic medication prescribing or deprescribing practices/patterns, or facilitators, or barriers in an acute care setting | The study describes antipsychotic medication prescribing or deprescribing practices/patterns, or facilitators, or barriers in either an outpatient setting, long-term care facility or nursing home |
| The study’s outcome(s) are any description of antipsychotic prescribing practices (prescribing or deprescribing)  The study’s outcome(s) are any perspectives, or experiences of patients, family members or healthcare providers of hospitalized patients with antipsychotic medication prescribing or deprescribing | The study’s outcome is something other than any perspectives, or experiences or impacts on patients, family members or healthcare providers of hospitalized patients |
|  |  |

**
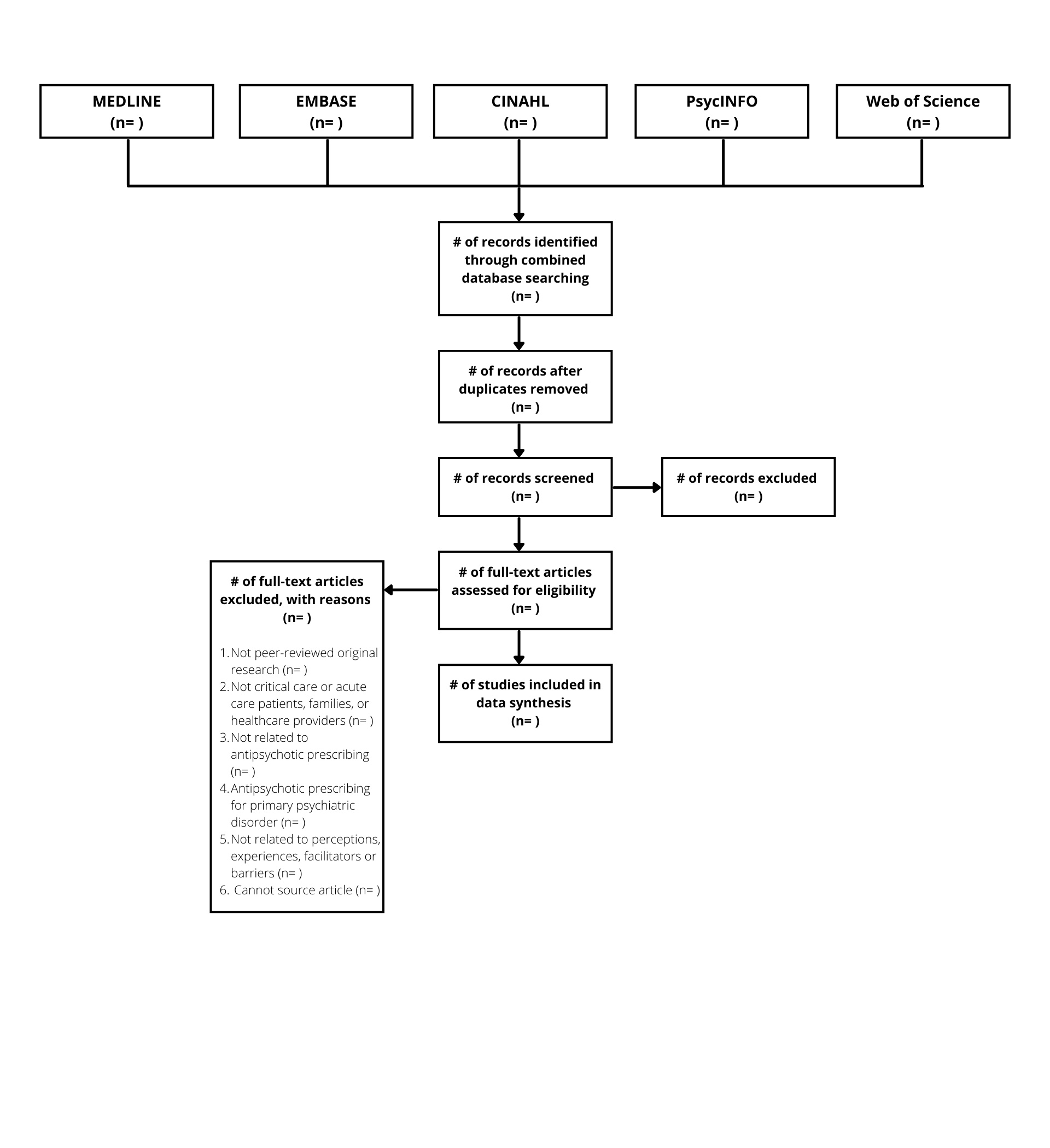
**

**Figure 1.** PRISMA flow exclusion diagram

**Appendix 1.** Database search strategies

# MEDLINE search strategy

1 exp antipsychotic agents/

2 (antipsychotic* or anti-psychotic* or neuroleptic* or psychotropic* or haldol or haloperidol or quetiapine or seroquel or risperidone or risperidal or olanzapine or zyprexa or methotrimeprazine or nozinan or ziprasidone or zeldox or geodon or aripiprazole or abilify).ti,ab,kf.

3. or/1-2

4 exp attitude of health personnel/

5 exp attitude to health/

6 exp health knowledge, attitudes, practice/

7 (knowledge adj2 attitude* adj2 perception*).ti,ab,kf.

8 (knowledge adj2 attitude* adj2 practice*).ti,ab,kf.

9 (attitude* or stance* or opinion* or insight* or percepti* or belie* or facilitator* or facilitat* or experience* or perspective* or barrier* or challeng*).ti,ab,kf.

10 or/4-9

11 exp critical illness/

12 exp intensive care units/

13 exp critical care/

14 exp hospitalization/

15 exp inpatients/

16 exp hospitals/

17 (critical care* or critical ill* or critically ill* or intensive care* or intensive care unit* or ICU* or inpatient* or hospitaliz* or admit* or admission* or hospital*).ti,ab,kf.

18 or/11-17

19 exp Practice Patterns, Physicians/

20 exp Drug Prescriptions/

21 (deprescrib* or deprescrip* or de-prescrib* or de-prescrip* or discontinu* or dis-continu* or deadopt* or de-adopt* or de-implement* or deimplement* or prescrib* or prescrip* or practic*).ti,ab,kf.

22 (prescri* adj2 practice*).ti,ab,kf.

23 (prescri* adj2 pattern*).ti,ab,kf.

24 or/19-23

25 3 and 10 and 18 and 24

# EMBASE search strategy

1 exp neuroleptic agent/

2 (antipsychotic* or anti-psychotic* or neuroleptic* or psychotropic* or haldol or haloperidol or quetiapine or seroquel or risperidone or risperidal or olanzapine or zyprexa or methotrimeprazine or nozinan or ziprasidone or zeldox or geodon or aripiprazole or abilify).ti,ab,kw.

3 or/1-2

4 exp health personnel attitude/

5 exp attitude to health/

6 (knowledge adj2 attitude* adj2 perception*).ti,ab,kw.

7 (knowledge adj2 attitude* adj2 practice*).ti,ab,kw.

8 (attitude* or stance* or opinion* or insight* or percepti* or belie* or facilitator* or facilitat* or experience* or perspective* or barrier* or challeng*).ti,ab,kw.

9 or/4-9

10 exp critical illness/

11 exp intensive care unit/

12 exp intensive care/

13 exp hospitalization/

14 exp hospital patient/

15 exp hospital/

16 exp hospital care/

17 (critical care* or critical ill* or critically ill* or intensive care* or intensive care unit* or ICU* or inpatient* or hospitaliz* or admit* or admission* or hospital*).ti,ab,kw.

18 or/11-17

19 exp clinical practice/

20 exp prescription/

21 (deprescrib* or deprescrip* or de-prescrib* or de-prescrip* or discontinu* or dis-continu* or deadopt* or de-adopt* or de-implement* or deimplement* or prescrib* or prescrip* or practic*).ti,ab,kw.

22 (prescri* adj2 practice*).ti,ab,kw.

23 (prescri* adj2 pattern*).ti,ab,kw.

24 or/19-23

25 3 and 10 and 18 and 24

# PsycINFO search strategy

1 exp neuroleptic drugs/

2 (antipsychotic* or anti-psychotic* or neuroleptic* or psychotropic* or haldol or haloperidol or quetiapine or seroquel or risperidone or risperidal or olanzapine or zyprexa or methotrimeprazine or nozinan or ziprasidone or zeldox or geodon or aripiprazole or abilify).ti,ab.

3 1 or 2

4 exp Health Attitudes/

5 exp Health Personnel Attitudes/

6 exp Attitudes/

7 exp Knowledge Level/

8 (knowledge adj2 attitude* adj2 perception*).ti,ab.

9 (knowledge adj2 attitude* adj2 practice*).ti,ab.

10 (attitude* or stance* or opinion* or insight* or percepti* or belie* or facilitator* or facilitat* or experience* or perspective* or barrier* or challeng*).ti,ab.

11 4 or 5 or 6 or 7 or 8 or 9 or 10

12 exp Intensive Care/

13 exp Hospitalization/

14 exp Hospitalized Patients/

15 exp Hospitals/

16 (critical care* or critical ill* or critically ill* or intensive care* or intensive care unit* or inpatient* or hospitaliz* or admit* or admission* or hospital*).ti,ab.

17 12 or 13 or 14 or 15 or 16

18 exp “Prescribing (Drugs)”/

19 exp Clinical Practice/

20 (deprescribe* or deprescrip* or de-prescrib* or de-prescrip* or discontinu* or dis-continu* or deadopt* or de-adopt* or de-implement* or deimplement or prescrib* or prescript* or practic*).ti,ab.

21 (prescri* adj2 practice*).ti,ab.

22 (prescri* adj2 pattern*).ti,ab.

23 18 or 19 or 20 or 21 or 22

24 3 and 8 and 14 and 23

# CINAHL search strategy

S1 (MH “Antipsychotic Agents+”)

S2 TI “antipsychotic*” or AB “antipsychotic*” or TI “anti-psychotic*” or AB “anti-psychotic*” or TI “neuroleptic*” or AB “neuroleptic*” or TI “psychotropic*” or AB “psychotropic*” or TI “Haldol” or AB “Haldol” or TI “haloperidol” or AB “haloperidol” or TI “quetiapine” or AB “quetiapine” or TI “seroquel” or AB “seroquel” or TI “risperidone” or AB “risperidone” or TI “risperidal” or AB “risperidal” or TI “olanzapine” or AB “olanzapine” or TI “Zyprexa” or AB “Zyprexa” or TI “methotrimeprazine” or AB “methotrimeprazine” or TI “nozinan” or AB “nozinan” or TI “ziprasidone” or AB “ziprasidone” or TI “zeldox” or AB “zeldox” or TI “Geodon” or AB “Geodon” or TI “aripiprazole” or AB “aripiprazole” or TI “abilify” or AB “abilify”

S3 S1 or S2

S4 (MH “Attitude of Health Personnel+”) or (MH “Attitude to Health+”) or (MH “Attitude to Illness+”) or (MM “Health Knowledge”)

S5 TI “attitude*” or AB “attitude*” or TI “stance*” or AB “stance*” or TI “opinion*” or AB “opinion*” or TI “insight*” or AB “insight*” or TI “percepti*” or AB “percepti” or TI “belie*” of AB “belie” or TI “facilitator*” or AB “facilitator” or TI “experience*” or AB “experience*” or TI “perspective*” or AB “perspective*” or TI “barrier*” or AB “barrier*” or TI “challeng*” or AB “challeng*”

S6 TI “Knowledge N2 attitude* N2 perception*” or AB “Knowledge N2 attitude* N2 perception*”

S7 TI “Knowledge N2 attitude* N2 practice*” or AB “Knowledge N2 attitude* N2 practice*”

S8 S4 or S5 or S6 or S7

S9 (MM “Critical Illness”) or (MM “Critically Ill Patients”)

S10 (MH “Critical Care+”) or (MH “Intensive Care Units+”)

S11 (MM “Inpatients”) or (MH “Patients+”)

S12 (MH “Hospitals+”) or (MH “Hospitalization+”)

S13 TI “critical care*” or AB “critical care*” or TI “critical ill*” or AB “critical ill*” or TI “critically ill*” or AB “critically ill*” or TI “intensive care*” or AB “intensive care*” or TI “intensive care unit*” or AB “intensive care unit*” or TI “inpatient*” or AB “inpatient*” or TI “hospitaliz*” or AB “hospitaliz*” or TI “admit*” or AB “admit*” or TI “admission*” or AB “admission*” or TI “hospital*” or AB “hospital*”

S14 S9 or S10 or S11 or S12 or S13

S15 (MM “Practice Patterns”) or (MM “Prescribing Patterns”) or (MH “Medical Practice+”) or (MM “Inappropriate Prescribing”)

S16 (MM “Deprescribing”)

S17 TI “deprescrib*” or AB “deprescrib*” or TI “deprescrip*” or AB “deprescrip*” or TI “de-prescrib*” or AB “de-prescib*” or TI “de-prescrip*” or AB “de-prescrip*” or TI “discontinu*” or AB “discontinue*” or TI “dis-continu*” or AB “dis-continu*” or TI “deadopt*” or AB “deadopt*” or TI “de-adopt*” or AB “de-adopt*” or TI “de-implement*” or AB “de-implement*” or TI “deimplement*” or AB “deimplement*” or TI “prescrib*” or AB “prescrib*” or TI “prescrip*” or AB “prescrip*” or TI “practic*” or AB “practic*”

S18 TI “Prescri* N2 practice*” or AB “Prescri* N2 practice*”

S19 TI “Prescri* N2 pattern*” or AB “Prescri* N2 pattern*”

S20 S15 or S16 or S17 or S18 or S19

S21 S3 and S7 and S12 and S20

# Web of Science search strategy

#1 TI=(antipsychotic* or anti-psychotic* or neuroleptic* or psychotropic* or haldol or haloperidol or quetiapine or seroquel or risperidone or risperidal or olanzapine or zyprexa or methotrimeprazine or nozinan or ziprasidone or zeldox or geodon or aripiprazole or abilify) or AB=(antipsychotic* or anti-psychotic* or neuroleptic* or psychotropic* or haldol or haloperidol or quetiapine or seroquel or risperidone or risperidal or olanzapine or zyprexa or methotrimeprazine or nozinan or ziprasidone or zeldox or geodon or aripiprazole or abilify)

#2 TI=(attitude of health personnel* or attitude* to health or health knowledge) or AB=(attitude of health personnel* or attitude* to health or health knowledge)

#3 TI=(knowledge NEAR/2 attitude* NEAR/2 perception*) or AB=(knowledge NEAR/2 attitude* NEAR/2 perception*)

#4 TI=(knowledge NEAR/2 attitude* NEAR/2 practice*) or AB=(knowledge NEAR/2 attitude* NEAR/2 practice*)

#5 TI=(attitude* or stance* or opinion* or insight* or percepti* or belie* or facilitator* or experience* or perspective* or barrier* or challeng*) or AB=(attitude* or stance* or opinion* or percepti* or belie* or facilitator* or experience* or perspective* or barrier* or challeng*)

#6 #2 OR #3 OR #4 or #5

#7 TI=(critical care* or critical ill* or critically ill* or intensive care* or intensive care unit* or ICU* or inpatient* or hospitaliz* or admit* or admission* or hospital*) or AB=(critical care* or critical ill* or critically ill* or intensive care* or intensive care unit* or ICU* or inpatient* or hospitaliz* or admit* or admission* or hospital*)

#8 TI=(deprescrib* or deprescrip* or de-prescrib* or de-prescrip* or discontinu* or dis-continu* or deadopt* or de-adopt* or de-implement* or deimplement* or prescrib* or prescrip* or practice*) or AB=( deprescrib* or deprescrip* or de-prescrib* or de-prescrip* or discontinu* or dis-continu* or deadopt* or de-adopt* or de-implement* or deimplement* or prescrib* or prescrip* or practic*)

#9 TI=(prescri* NEAR/2 practice*) or AB=(prescri* NEAR/2 practice*)

#10 TI=( prescri* NEAR/2 pattern*) or AB=(prescri* NEAR/2 pattern*)

#11 #8 or #9 or #10

#12 #1 AND #6 AND #7 AND #11
