# Supplementary material for: A scoping review of perceptions from healthcare professionals on antipsychotic prescribing practices in acute care settings
Source: BMC Health Serv Res. 2022 Oct 21;22:1272. doi: 10.1186/s12913-022-08650-7 (PMC9587627; doi:10.1186/s12913-022-08650-7)
Supplement: Supplementary file 2 — Additional file 2. [file 12913_2022_8650_MOESM2_ESM.docx]

**Online Appendix 1.** Pre-specified antipsychotic medications evaluated in search strategy

| **Selected antipsychotic medications for evaluation** |
| --- |
| Haloperidol/Haldol® |
| Quetiapine/Seroquel® (immediate release and extended release) |
| Risperidone/Risperidal® (immediate release and extended release) |
| Ziprasidone/Zeldox®/Geodon® |
| Aripiprazole/Abilify® |
| Olanzapine/Zyprexa® |
| Methotrimeprazine/Nozinan® |

**Online Appendix 2.** Alphabetical references for included studies [1-80]

1. Al-Qadheeb, N.S., et al., *Antipsychotic prescribing patterns, and the factors and outcomes associated with their use, among patients requiring prolonged mechanical ventilation in the long-term acute care hospital setting.* Annals of Pharmacotherapy, 2013. **47**(2): p. 181-8.

2. Almehairi, E., et al., *Antipsychotics (APs) prescribing in critically ill delirious patients, the reported versus the perceived practice.* Critical Care. Conference: 38th International Symposium on Intensive Care and Emergency Medicine, ISICEM, 2018. **22**(Supplement 1).

3. Basciotta, M. and S.J. Herzig, *Risk of in-hospital mortality or cardiopulmonary arrest associated with antipsychotic use.* Journal of General Internal Medicine, 2018. **33 (2 Supplement 1)**: p. 337-338.

4. Bascom, P.B., J.L. Bordley, and A.J. Lawton, *High-dose neuroleptics and neuroleptic rotation for agitated delirium near the end of life.* American Journal of Hospice & Palliative Medicine, 2014. **31**(8): p. 808-11.

5. Bedouch, P., et al., *Trends in pharmacists' medication order review in French hospitals from 2006 to 2009: Analysis of pharmacists' interventions from the Act-IP© website observatory.* Journal of Clinical Pharmacy and Therapeutics, 2015. **40**(1): p. 32-40.

6. Bervoets, C., et al., *Prescribing preferences in rapid tranquillisation: a survey in Belgian psychiatrists and emergency physicians.* BMC Research Notes, 2015. **8**: p. 218.

7. Biringen, E.K., et al., *Psychotropic medications in oncology.* Supportive Care in Cancer., 2021.

8. Boncyk, C.S., et al., *Pharmacologic Management of Intensive Care Unit Delirium: Clinical Prescribing Practices and Outcomes in More Than 8500 Patient Encounters.* Anesth Analg, 2021. **133**(3): p. 713-722.

9. Brennan, M.J., et al., *Acute care for elders (ACE) program lowers new antipsychotic prescription rate.* Journal of the American Geriatrics Society, 2018. **66 (Supplement 2)**: p. S119.

10. Brett, J., et al., *Off‐label quetiapine prescribing in general hospital inpatients: an Australian experience.* Journal of Pharmacy Practice & Research, 2020. **50**(4): p. 316-320.

11. Briskman, I., R. Dubinski, and Y. Barak, *Treating delirium in a general hospital: a descriptive study of prescribing patterns and outcomes.* Int Psychogeriatr, 2010. **22**(2): p. 328-31.

12. Brown, G. and W. Scott, *An assessment of a sedative algorithm for sleep in an intensive care unit.* Official journal of the Canadian Association of Critical Care Nurses / CACCN, 1998. **9**(4): p. 20-24.

13. Campillo, A., et al., *The B52 combination is not frequently used in emergency departments and causes a high proportion of patients to fall asleep.* Annals of Emergency Medicine, 2012. **60**(4): p. S147.

14. Ceraso, D.H., et al., *Latin American survey on delirium in critical patients. [Spanish].* Medicina Intensiva, 2010. **34**(8): p. 495-505.

15. Chan, E.W., et al., *Management of acute agitation in the accident and emergency setting: A survey of practice in Hong Kong.* EMA - Emergency Medicine Australasia, 2015. **27**: p. 31.

16. Chan, E.W., et al., *Variation in the management of hypothetical cases of acute agitation in Australasian emergency departments.* EMA - Emergency Medicine Australasia, 2011. **23**(1): p. 23-32.

17. Chawla, R., *ISCCM-MARS-mobilization-analgesiarelaxant-sedation survey-preliminary findings.* Indian Journal of Critical Care Medicine, 2013. **17**: p. 2-3.

18. Collet, M.O., T. Thomsen, and I. Egerod, *Nurses' and physicians' approaches to delirium management in the intensive care unit: A focus group investigation.* Australian critical care : official journal of the Confederation of Australian Critical Care Nurses, 2019. **32**(4): p. 299-305.

19. Costa-Dias, M.J., et al., *Medication fall risk in old hospitalized patients: a retrospective study.* Nurse Education Today, 2014. **34**(2): p. 171-6.

20. Cowling, M., et al., *Characterizing the role of haloperidol for analgesia in the emergency department.* Journal of Pain Management, 2019. **12**(2): p. 141-146.

21. D'Angelo, R.G., et al., *Impact of an Antipsychotic Discontinuation Bundle During Transitions of Care in Critically Ill Patients.* Journal of Intensive Care Medicine, 2019. **34**(1): p. 40-47.

22. Debacker, J., et al., *Sedation practice in extracorporeal membrane oxygenation- treated patients with acute respiratory distress syndrome: A retrospective study.* ASAIO Journal, 2018. **64**(4): p. 544-551.

23. Devlin, J.W., et al., *Current perceptions and practices surrounding the recognition and treatment of delirium in the intensive care unit: a survey of 250 critical care pharmacists from eight States.* Annals of Pharmacotherapy, 2011. **45**(10): p. 1217-1229.

24. Dyal, S. and R. MacLaren, *The Assessment and Management Practices of Acute Alcohol Withdrawal: Results of a Nationwide Survey of Critical Care Pharmacists.* Hospital Pharmacy, 2019. **54**(1): p. 22-31.

25. Dzierba, A.L., et al., *Current practice and perceptions regarding pain, agitation and delirium management in patients receiving venovenous extracorporeal membrane oxygenation.* Journal of Critical Care, 2019. **53**: p. 98-106.

26. Eastwood, G.M., et al., *A questionnaire survey of critical care nurses' attitudes to delirium assessment before and after introduction of the CAM-ICU.* Australian Critical Care, 2012. **25**(3): p. 162-169.

27. Ely, E.W., et al., *Current opinions regarding the importance, diagnosis, and management of delirium in the intensive care unit: a survey of 912 healthcare professionals.* Critical Care Medicine, 2004. **32**(1): p. 106-12.

28. Farrokh, S., et al., *Continuation Rate of Atypical Antipsychotics After Discharge When Initiated in the Intensive Care Unit.* J Pharm Pract, 2017. **30**(3): p. 342-346.

29. Flores, D.J., et al., *Nursing practices and perceptions towards delirium in the burn intensive care unit.* Journal of Burn Care and Research, 2015. **36**: p. S156.

30. Fontaine, G.V., et al., *Newly Initiated In-Hospital Antipsychotics Continued at Discharge in Non-psychiatric Patients.* Hospital Pharmacy, 2018. **53**(5): p. 308-315.

31. Gilani, A., et al., *Delirium management by medical professionals in intensive care-a national survey.* Journal of the Intensive Care Society, 2020. **21 (2 SUPPL)**: p. 219-220.

32. Gilbert, B., et al., *Evaluation of Neuroleptic Utilization in the Intensive Care Unit During Transitions of Care.* J Intensive Care Med, 2017. **32**(2): p. 158-162.

33. Gill, K.V., et al., *Sedation practices in adult intensive care unit (ICU) patients on mechanical ventilation.* Critical Care Medicine, 2009. **37 (12 SUPPL.)**: p. A482.

34. Glass, M. and W. Gibson, *Clinical impact of pharmacist assessment in patients with ICU delirium.* Critical Care Medicine, 2018. **46 (Supplement 1)**: p. 460.

35. Gong, Z.P., et al., *Survey of attitudes and behaviors of healthcare professionals on delirium in ICU.* Chinese Journal of Traumatology - English Edition, 2009. **12**(6): p. 328-333.

36. Herzig, S.J., et al., *Antipsychotic medication utilization in nonpsychiatric hospitalizations.* J Hosp Med, 2016. **11**(8): p. 543-9.

37. Hosie, A., et al., *Clinicians' delirium treatment practice, practice change, and influences: A national online survey.* Palliative Medicine 2021. **35**(8): p. 1553-1563.

38. Hui, D., et al., *Neuroleptic prescription pattern for delirium in patients with advanced cancer.* J Palliat Care, 2011. **27**(2): p. 141-147.

39. Johnson, K., et al., *Early recognition of delirium in trauma patients.* Intensive & Critical Care Nursing, 2016. **34**: p. 28-32.

40. Kim, D.H., et al., *Longitudinal Trends and Variation in Antipsychotic Use in Older Adults After Cardiac Surgery.* J Am Geriatr Soc, 2018. **66**(8): p. 1491-1498.

41. Kloet, M.A., et al., *Prospective Assessment of Inpatient Boxed Warning Prescriber Adherence.* Journal of patient safety, 2017. **13**(1): p. 25-30.

42. Kotfis, K., et al., *Multicenter assessment of sedation and delirium practices in the intensive care units in Poland - is this common practice in Eastern Europe?* BMC Anesthesiology, 2017. **17**(1): p. 1-10.

43. Kram, B., S. Kram, and K. Brooks, *Implications of atypical antipsychotic prescribing in the intensive care unit.* Critical Care Medicine, 2014. **42**(12): p. A1512-A1513.

44. Kram, B.L., et al., *A Pharmacy-Based Electronic Handoff Tool to Reduce Discharge Prescribing of Atypical Antipsychotics Initiated in the Intensive Care Unit: A Quality Improvement Initiative.* Journal of Pharmacy Practice, 2019. **32**(4): p. 434-441.

45. Kuscu, M.K., et al., *Attitudes and priorities of training clinicians in diagnosing delirium in an academic hospital.* Marmara Medical Journal, 2004. **17**(3): p. 99-104.

46. Levine, A.R., et al., *Risk Factors for Continuation of Atypical Antipsychotics at Hospital Discharge in Two Intensive Care Unit Cohorts.* Clinical Medicine Insights: Psychiatry, 2019. **10**: p. 1179557319863813.

47. Loh, E.C. and C.N. Chin, *The use of orodispersible olanzapine in the management of terminal delirium in palliative care.* European Neuropsychopharmacology, 2011. **21**: p. S611-S612.

48. Loh, K.P., et al., *Long-term outcomes of elders discharged on antipsychotics.* Journal of Hospital Medicine, 2016. **11**(8): p. 550-555.

49. Marshall, J., et al., *Antipsychotic utilization in the intensive care unit and in transitions of care.* J Crit Care, 2016. **33**: p. 119-124.

50. Masman, A., et al., *Medication use during end-of-life care in a palliative care centre.* International Journal of Clinical Pharmacy, 2015. **37**(5): p. 767-775.

51. Mattison, M.L., et al., *A standardized, bundled approach to providing geriatric-focused acute care.* Journal of the American Geriatrics Society, 2014. **62**(5): p. 936-942.

52. McNeill, R., et al., *Polypharmacy in Palliative Care: Two Deprescribing Tools Compared with a Clinical Review.* Journal of Palliative Medicine, 2021. **24**(5): p. 661-667.

53. Meagher, D., et al., European Neuropsychopharmacology, 2013. **23**: p. S551-S552.

54. Mehta, S., et al., *A multicenter survey of Ontario intensive care unit nurses regarding the use of sedatives and analgesics for adults receiving mechanical ventilation.* Journal of Critical Care, 2007. **22**(3): p. 191-196.

55. Mo, Y., A.E. Zimmermann, and M.C. Thomas, *Practice patterns and opinions on current clinical practice guidelines regarding the management of delirium in the intensive care unit.* Journal of Pharmacy Practice, 2017. **30**(2): p. 162-171.

56. Palacios-Cena, D., et al., *How do doctors and nurses manage delirium in intensive care units? A qualitative study using focus groups.* BMJ Open, 2016. **6**(1):e009678.

57. Patel, M. and H. Groves, *Prescribing practices of pharmacologic agents used to promote sleep in the ICU.* Critical Care Medicine. Conference: 48th Critical Care Congress of the Society of Critical Care Medicine, SCCM, 2019. **47**(1 Supplement 1).

58. Patel, R.P., et al., *Delirium and sedation in the intensive care unit: survey of behaviors and attitudes of 1384 healthcare professionals.* Crit Care Med, 2009. **37**(3): p. 825-832.

59. Ranzani, O.T., et al., *Evaluation of a minimal sedation protocol using ICU sedative consumption as a monitoring tool: a quality improvement multicenter project.* Critical Care, 2014. **18**(4): p. 580-580.

60. Rhoney, D.H. and K.R. Murry, *National survey of the use of sedating drugs, neuromuscular blocking agents, and reversal agents in the intensive care unit.* J Intensive Care Med, 2003. **18**(3): p. 139-145.

61. Salluh, J.I., et al., *Delirium recognition and sedation practices in critically ill patients: a survey on the attitudes of 1015 Brazilian critical care physicians.* Journal of Critical Care, 2009. **24**(4): p. 556-562.

62. Selim, A.A. and E. Wesley Ely, *Delirium the under-recognised syndrome: survey of healthcare professionals' awareness and practice in the intensive care units.* Journal of Clinical Nursing, 2017. **26**(5-6): p. 813-824.

63. Silverman, D., et al., *Quetiapine use in the surgical intensive care unit.* Critical Care Medicine, 2013. **41**(12): p. A216.

64. Someya, T., et al., *A survey on the drug therapy for delirium.* Psychiatry and Clinical Neurosciences, 2001. **55**(4): p. 397-401.

65. Stuart, M.M., et al., *Pharmacist-driven discontinuation of antipsychotics for ICU delirium: A quasi-experimental study.* JACCP Journal of the American College of Clinical Pharmacy, 2020. **3**(6): p. 1009-1014.

66. Swan, J.T., et al., *Antipsychotic use and diagnosis of delirium in the intensive care unit.* Crit Care, 2012. **16**(3): p. R84.

67. Sweeney, R.M., et al., *A national survey of the management of delirium in UK intensive care units.* Qjm, 2010. **103**(4): p. 243-251.

68. Sztrymf, B., et al., *Diagnosis and management of delirium in critical care patients: A French national survey.* Reanimation, 2012. **21**(5): p. 557-562.

69. Thacker, S., *Junior doctors and emergency tranquillisation of elderly, confused patients: A survey.* Psychiatric Bulletin, 1996. **20**(4): p. 212-214.

70. Thiboutot, Z., et al., *Antipsychotic drug use and screening for delirium in mechanically ventilated patients in canadian intensive care units: An observational study.* Canadian Journal of Hospital Pharmacy, 2016. **69**(2): p. 107-113.

71. Tomichek, J.E., et al., *Antipsychotic prescribing patterns during and after critical illness: a prospective cohort study.* Crit Care, 2016. **20**(1): p. 378.

72. Trenaman, S.C., et al., *Antipsychotic Drug Dispensations in Older Adults, Including Continuation After a Fall-Related Hospitalization: Identifying Adherence to Screening Tool of Older Persons' Potentially Inappropriate Prescriptions Criteria Using the Nova Scotia Seniors' Pharmacare Program and Canadian Institute for Health's Discharge Databases.* Current Therapeutic Research, 2018. **89**: p. 27-36.

73. Trogrlic, Z., et al., *Current practices in ICU delirium management: A prospective multicenter study in the Netherlands.* Critical Care, 2013. **17**: p. S148.

74. Trogrlic, Z., et al., *Current perspectives, beliefs and practices concerning delirium in critically ill patients: A multicenter survey among Dutch healthcare professionals.* Critical Care, 2013. **17**: p. S148-S149.

75. Tropea, J., et al., *Use of antipsychotic medications for the management of delirium: an audit of current practice in the acute care setting.* Int Psychogeriatr, 2009. **21**(1): p. 172-179.

76. van den Boogaard, M., et al., *Implementation of a delirium assessment tool in the ICU can influence haloperidol use.* Crit Care, 2009. **13**(4): p. R131.

77. Wang, J., et al., *A National Multicenter Survey on Management of Pain, Agitation, and Delirium in Intensive Care Units in China.* Chinese Medical Journal, 2017. **130**(10): p. 1182-1188.

78. Weir, D.L., et al., *Both New and Chronic Potentially Inappropriate Medications Continued at Hospital Discharge Are Associated With Increased Risk of Adverse Events.* Journal of the American Geriatrics Society, 2020. **68**(6): p. 1184-1192.

79. Wong, A., et al., *Prescribing patterns and safety of intramuscular olanzapine in hospitalized elderly patients.* Canadian Journal of Hospital Pharmacy, 2014. **67 (1)**: p. 79.

80. Yasuyuki, O., et al., *Expert opinions on the first-line pharmacological treatment for delirium in Japan: a conjoint analysis.* International Psychogeriatrics, 2016. **28**(6): p. 1041-1050.
