# Supplementary material for: A scoping review of perceptions from healthcare professionals on antipsychotic prescribing practices in acute care settings
Source: BMC Health Serv Res. 2022 Oct 21;22:1272. doi: 10.1186/s12913-022-08650-7 (PMC9587627; doi:10.1186/s12913-022-08650-7)
Supplement: Supplementary file 3 — Additional file 3: Supplementary Table 1. Search strategy used in MEDLINE. Supplementary Table 2. Characteristics of included studies. Supplementary Table 3. Antipsychotic reported outcomes of included studies. Supplementary Table 4. Reportedantipsychotic medication prescribing indications included studies by acute care setting. Supplementary Table 5. Measured and perceived antipsychotics prescribed and prescribing indications reported for included studies, by acutecare setting. Supplementary Table 6. Number of studies reporting on healthcare professional reported perceived antipsychotic prescribing practices in acute care, by acute care setting and antipsychotic type. Supplementary Table 7. Number of studies reporting on measured outcomes of antipsychotic prescribing practices in the acute care setting, by acute care setting and antipsychotic type. Supplementary Table 8. Reported additionally prescribed sedative hypnotic medications for included studies reporting on antipsychotic medication prescribing, by acute care setting. Supplementary Table 9. Reported co-prescribed sedative hypnotic medications with antipsychotic medications for included studies which report on additionally prescribed medications, by acute care setting. Supplementary Table 10. Domains and constructs according to the Theoretical Domains Framework of perspectives on antipsychotic prescribing from healthcare professionals for included studies, by acute care setting. Supplementary Table 11. Deductive thematic analysis using the Theoretical Domains Framework on perceptions on antipsychotic prescribing forincluded studies. Supplementary Table 12. Description of reported antipsychotic deprescribing strategies applied in parallel for included studies reporting on antipsychotic medication prescribing. [file 12913_2022_8650_MOESM3_ESM.docx]

**Supplementary Figures and Tables**

**
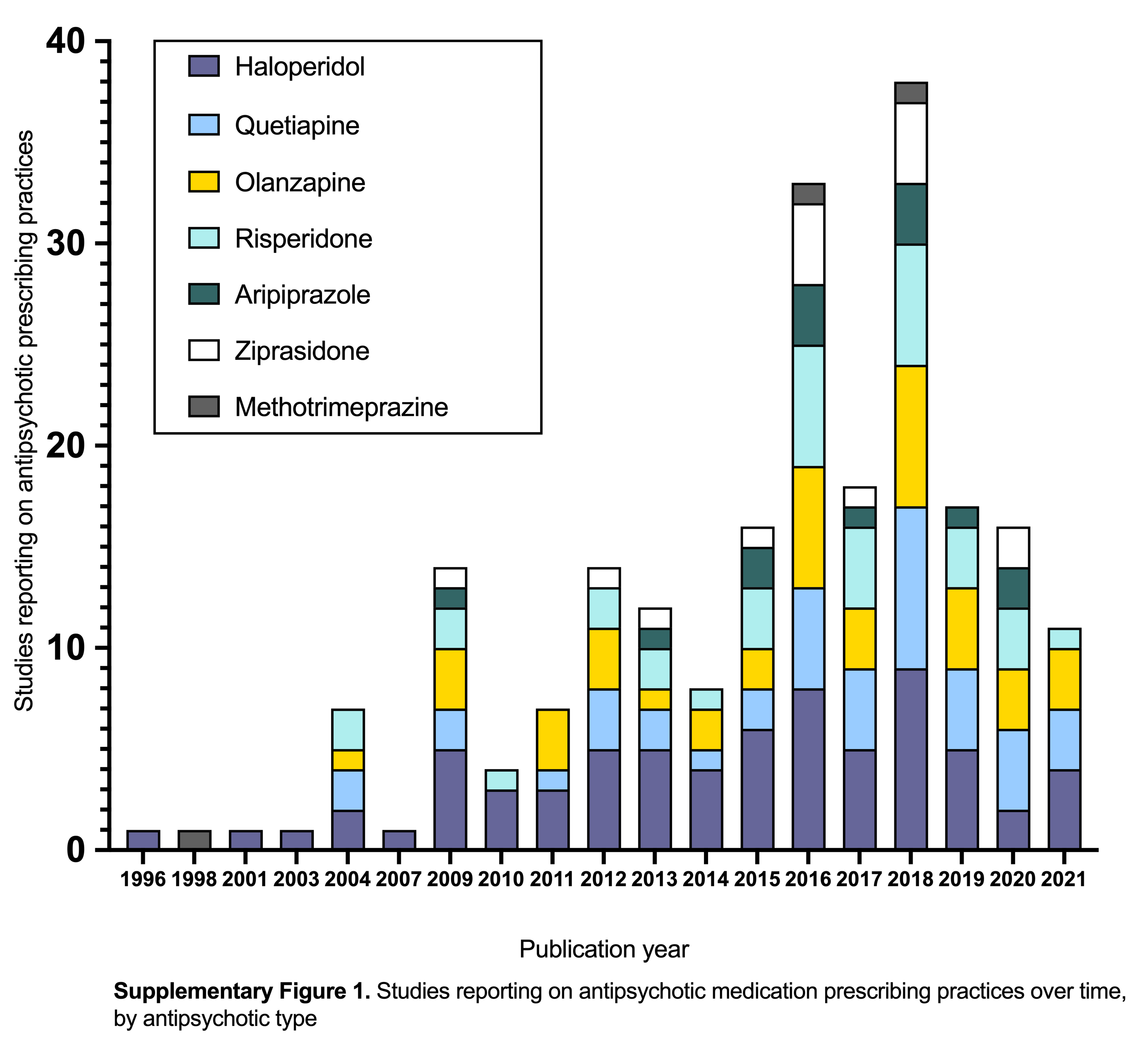
**

**
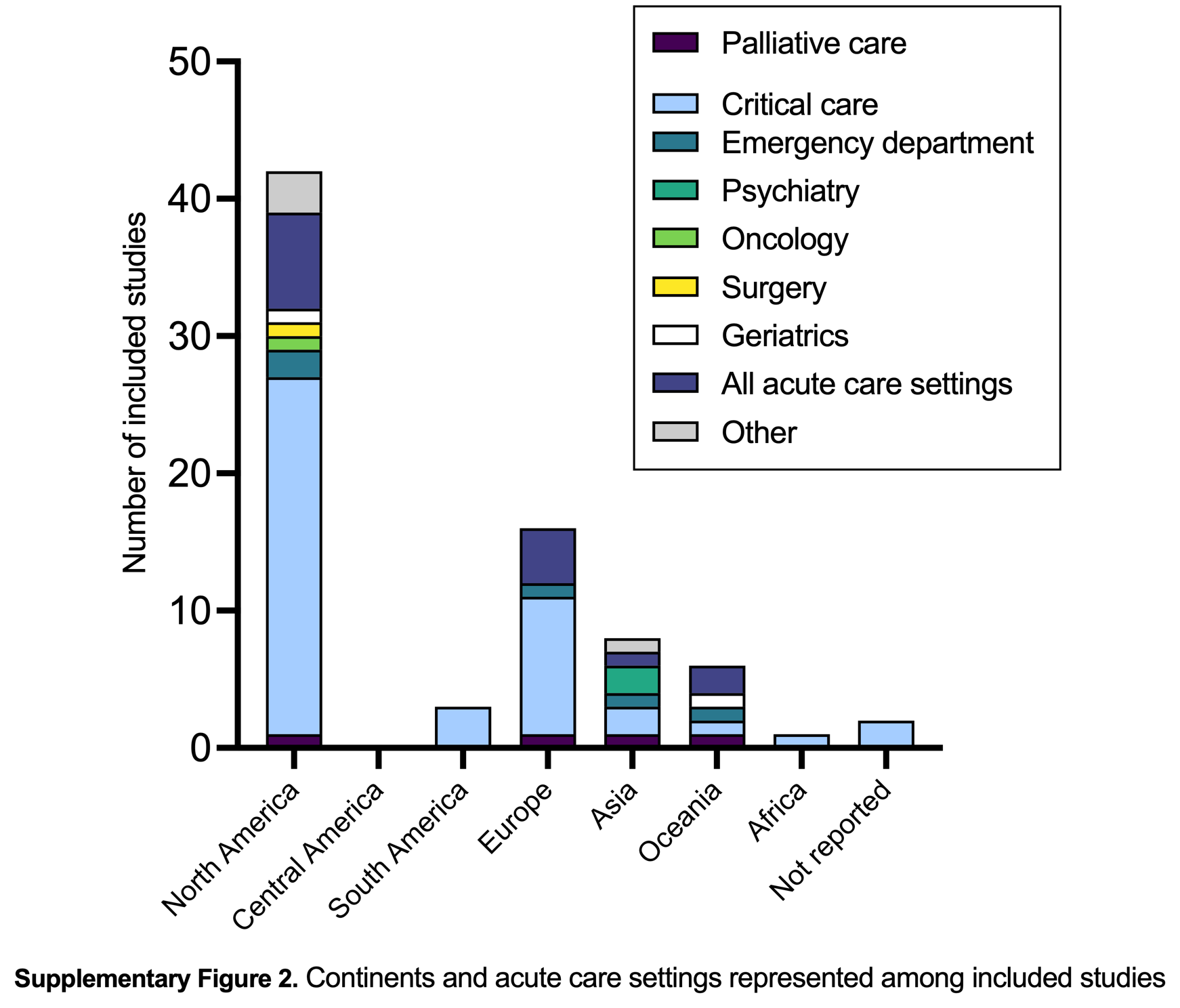
**

**Supplementary Table 1.** Search strategy used in MEDLINE

| **Search component** | **Search terms** |
| --- | --- |
| Antipsychotic medications | 1 exp antipsychotic agents/  2 (antipsychotic* or anti-psychotic* or neuroleptic* or psychotropic* or haldol or haloperidol or quetiapine or seroquel or risperidone or risperidal or olanzapine or zyprexa or methotrimeprazine or nozinan or ziprasidone or zeldox or geodon or aripiprazole or abilify).ti,ab,kf.  3 or/1-2 |
| Perspectives | 4 exp attitude of health personnel/  5 exp attitude to health/  6 exp health knowledge, attitudes, practice/  7 (knowledge adj2 attitude* adj2 perception*).ti,ab,kf.  8 (knowledge adj2 attitude* adj2 practice*).ti,ab,kf.  9 (attitude* or stance* or opinion* or insight* or percepti* or belie* or facilitator* or facilitat* or experience* or perspective* or barrier* or challeng*).ti,ab,kf.  10 or/4-9 |
| Acute care setting | 11 exp critical illness/  12 exp intensive care units/  13 exp critical care/  14 exp hospitalization/  15 exp inpatients/  16 exp hospitals/  17 (critical care* or critical ill* or critically ill* or intensive care* or intensive care unit* or ICU* or inpatient* or hospitaliz* or admit* or admission* or hospital*).ti,ab,kf.  18 or/11-17 |
| Prescribing practices | 19 exp Practice Patterns, Physicians/  20 exp Drug Prescriptions/  21 (deprescrib* or deprescrip* or de-prescrib* or de-prescrip* or discontinu* or dis-continu* or deadopt* or de-adopt* or de-implement* or deimplement* or prescrib* or prescrip* or practic*).ti,ab,kf.  22 (prescri* adj2 practice*).ti,ab,kf.  23 (prescri* adj2 pattern*).ti,ab,kf.  24 or/19-23 |
| All | 25 3 and 10 and 18 and 24 |

No limiters or restrictions were applied to any database searches.

**Supplementary Table 2.** Characteristics of included studies

| First author | Year |  |  | Research type | Study type | Country/Continent | Clinical specialty | Population | Number of included patients | Number of included healthcare professionals |
| --- | --- | --- | --- | --- | --- | --- | --- | --- | --- | --- |
| Intensive Care | | | | | | | | | | |
| Almehairi, E. | 2018 |  |  | Observational | Cross-sectional survey + chart review | United Kingdom | Intensive care | Patients and professionals | 188 | 43 |
| Boncyk, C.S. | 2021 |  |  | Observational | Retrospective cohort study | United States | Intensive care | Patients | 7,879 |  |
| Brown, G. | 1998 |  |  | Interventional | Quasi-experimental study | Canada | Intensive care | Patients | 78 |  |
| Collet, M.O. | 2019 |  |  | Observational | Focus groups | Denmark | Intensive care | Professionals |  | 39 |
| D’Angelo, R.G. | 2019 |  |  | Interventional | Pre-post interventional study | United States | Intensive care | Patients | 281 |  |
| DeBacker, J. | 2018 |  |  | Observational | Retrospective cohort study | Canada | Intensive care | Patients | 45 |  |
| Devlin, J.W. | 2011 |  |  | Observational | Cross-sectional survey | United States | Intensive care | Professionals |  | 250 |
| Dyal, S. | 2019 |  |  | Observational | Cross-sectional survey | United States | Intensive care | Professionals |  | 94 |
| Dzierba, A.L. | 2019 |  |  | Observational | Cross-sectional survey | United States | Intensive care | Professionals |  | 221 |
| Eastwood, G.M. | 2012 |  |  | Interventional | Quality improvement study | Australia | Intensive care | Professionals |  | 174 |
| Ely, E.W. | 2004 |  |  | Observational | Cross-sectional survey | United States | Intensive care | Professionals |  | 912 |
| Farrokh, S. | 2017 |  |  | Observational | Retrospective cohort study | United States | Intensive care | Patients | 100 |  |
| Flores, D.J. | 2015 |  |  | Observational | Cross-sectional survey | United States | Intensive care | Professionals |  | 41 |
| Gilbert, B. | 2017 |  |  | Observational | Retrospective cohort study | United States | Intensive care | Patients | 236 |  |
| Gill, K.V. | 2012 |  |  | Observational | Cross-sectional survey + retrospective cohort study | United States | Intensive care | Patients and professionals | 496 | 53 |
| Glass, M. | 2018 |  |  | Interventional | Pre-post interventional study | Not reported | Intensive care | Patients | 100 |  |
| Johnson, K. | 2016 |  |  | Interventional | Pre-post interventional study | United States | Intensive care | Professionals |  | 72 |
| Kim, D.H. | 2018 |  |  | Observational | Retrospective cohort study | United States | Cardiac surgery | Patients | 293,212 |  |
| Kloet, M.A. | 2017 |  |  | Interventional | Quality improvement study | United States | Intensive care | Patients | 393 |  |
| Kram, B.L. | 2015 |  |  | Observational | Retrospective cohort study | United States | Intensive care | Patients | 156 |  |
| Kram, B.L. | 2019 |  |  | Interventional | Quality improvement study | United States | Intensive care | Patients | 358 |  |
| Levine, A.R. | 2019 |  |  | Observational | Retrospective cohort study | United States | Intensive care | Patients | 279 |  |
| Marshall, J. | 2016 |  |  | Observational | Retrospective cohort study | United States | Intensive care | Patients | 39,246 |  |
| Mo, Y. | 2017 |  |  | Observational | Cross-sectional survey | United States | Intensive care | Professionals |  | 635 |
| Palacios-Ceña, D. | 2016 |  |  | Observational | Focus groups | Spain | Intensive care | Professionals |  | 38 |
| Patel, M. | 2019 |  |  | Observational | Retrospective cohort study | Not reported | Intensive care | Patients | 43 |  |
| Patel, R.P. | 2009 |  |  | Observational | Cross-sectional survey | United States | Intensive care | Professionals |  | 1,384 |
| Ranzani, O.T. | 2014 |  |  | Interventional | Quality improvement study | Brazil | Intensive care | Patients | 22,965 |  |
| Rhoney, D.H. | 2003 |  |  | Observational | Cross-sectional survey | United States | Intensive care | Professionals |  | 474 |
| Silverman, D. | 2013 |  |  | Observational | Retrospective cohort study | United States | Intensive care | Patients | 70 |  |
| Stuart, M.M. | 2020 |  |  | Interventional | Quasi-experimental study (retrospective) | United States | Intensive care | Patients | 158 |  |
| Swan, J.T. | 2012 |  |  | Observational | Retrospective cohort study | United States | Intensive care | Patients | 164,996 |  |
| Thiboutot, Z. | 2016 |  |  | Observational | Prospective cohort study | Canada | Intensive care | Patients | 712 |  |
| Tomichek, J.E. | 2016 |  |  | Observational | Prospective cohort study | United States | Intensive care | Patients | 500 |  |
| Trogrlic, Z. | 2013 |  |  | Observational | Prospective cohort study | Netherlands | Intensive care | Patients | 1,576 |  |
| van den Boogaard, M. | 2009 |  |  | Interventional | Quality improvement study | Netherlands | Intensive care | Patients | 1,742 |  |
| Inpatient | | | | | | | | | | |
| Al-Qadheeb, N.S. | 2013 |  |  | Observational | Prospective cohort study | United States | All acute care settings | Patients | 180 |  |
| Basciotta, M. | 2018 |  |  | Observational | Retrospective cohort study | United States | All acute care settings | Patients | 150,948 |  |
| Bascom, P.B. | 2014 |  |  | Observational | Case report or case series | USA | Palliative care | Patients | 2 |  |
| Bedouch, P. | 2015 |  |  | Observational | Cross-sectional survey | France | All acute care settings | Professionals |  | 201 |
| Birigen, E.K. | 2021 |  |  | Observational | Cross-sectional survey | United States | Oncology | Professionals |  | 65 |
| Brennan, M. | 2018 |  |  | Observational | Case-control | USA | Geriatrics | Patients | 1,570 |  |
| Brett, J. | 2020 |  |  | Observational | Retrospective cohort study + chart review | Australia | Geriatrics | Patients | 793 |  |
| Briskman, I. | 2010 |  |  | Observational | Retrospective cohort study | Israel | All acute care settings | Patients | 191 |  |
| Costa-Dias, M.J. | 2014 |  |  | Observational | Retrospective cohort study | Portugal | All acute care settings | Patients | 193 |  |
| Fontaine, G.V. | 2018 |  |  | Observational | Retrospective cohort study | United States | All acute care settings | Patients | 8,297 |  |
| Herzig, S.J. | 2016 |  |  | Observational | Retrospective cohort study | United States | All acute care settings | Patients | 2,695,081 |  |
| Hosie, A. | 2021 |  |  | Observational | Cross-sectional survey | Australia (All) | All acute care settings | Professionals |  | 475 |
| Hui, D. | 2011 |  |  | Observational | Retrospective cohort study | United States | Palliative care | Patients | 100 |  |
| Kuscu, M.K. | 2004 |  |  | Observational | Cross sectional survey + semi-structured interviews | Turkey | Internal medicine and surgery | Professionals |  | 75 |
| Loh, E.C. | 2011 |  |  | Observational | Case report or case series | Malaysia | Palliative care | Patients | 3 |  |
| Loh, K.P. | 2016 |  |  | Observational | Retrospective cohort study + chart review | United States | Internal medicine and surgery | Patients | 260 |  |
| Masman, A.D. | 2015 |  |  | Observational | Retrospective cohort study | Netherlands | Palliative care | Patients | 208 |  |
| Mattison, M.L.P. | 2014 |  |  | Interventional | Pre-post control interventional study | United States | All acute care settings | Patients | 19,949 |  |
| McNeill, R. | 2021 |  |  | Observational | Retrospective cohort study | New Zealand | Palliative care | Patients | 50 |  |
| Meagher, D. | 2013 |  |  | Observational | Cross-sectional survey | Europe^†^ | All acute care settings | Professionals |  | 200 |
| Someya, T. | 2001 |  |  | Observational | Cross-sectional study | Japan | Psychiatry | Patients | 167 |  |
| Thacker, S. | 1996 |  |  | Observational | Cross-sectional survey | United Kingdom | All acute care settings | Professionals |  | 46 |
| Trenaman, S.C. | 2018 |  |  | Observational | Cross-sectional study | Canada | All acute care settings | Patients | 585 |  |
| Tropea, J. | 2009 |  |  | Observational | Medical record audit | Australia | All acute care settings | Patients |  | 174 |
| Weir, D.L. | 2020 |  |  | Observational | Prospective cohort study | Canada | Internal medicine, cardiac and thoracic surgery | Patients | 2,402 |  |
| Wong, A. | 2014 |  |  | Observational | Retrospective cohort study | Canada | All acute care settings | Patients | 76 |  |
| Yasuyuki, O. | 2016 |  |  | Observational | Cross-sectional survey | Japan | Psychiatry | Professionals |  | 154 |
| Emergency department | | | | | | | | | | |
| Bervoets, C. | 2015 |  |  | Observational | Cross-sectional survey | Belgium | Emergency department | Professionals |  | 110 |
| Campillo, A. | 2012 |  |  | Observational | Retrospective cohort study | United States | Emergency department | Patients | 1,253 |  |
| Chan, E.W. | 2011 |  |  | Observational | Cross-sectional survey | Australia, New Zealand | Emergency department | Professionals |  | 783 |
| Chan, E.W. | 2015 |  |  | Observational | Cross-sectional survey | Hong Kong | Emergency department | Professionals |  | 281 |
| Cowling, M. | 2019 |  |  | Observational | Cross-sectional survey | United States | Emergency department | Professionals |  | 129 |

Organized in order of setting; author; then publication year

^†^United Kingdom, Netherlands, Italy, Switzerland, Germany, Spain, Portugal, Ireland, Sweden, Denmark, Austria

^‡^Argentina, Mexico, Chile, Colombia

**Supplementary Table 3.** Antipsychotic reported outcomes of included studies

| **Intensive care** | | | | | | | | | | | | | | | |
| --- | --- | --- | --- | --- | --- | --- | --- | --- | --- | --- | --- | --- | --- | --- | --- |
| First author | Year | Study type | Country/Continent | Reported or measured antipsychotic outcomes | | | | | | | | | |  | Conclusions* |
|  |  |  |  | Participant reported prescribing practices | Measured prescribing practices | Characterize monitoring and management of pain, agitation, or delirium | Measured prescribing practices at transitions of care | Antipsychotic deprescribing | Evaluation of Inappropriate antipsychotic prescribing practices | Delirium outcomes | Mortality | Sedation effects | Prescribing practice audit | Falls |  |
| Almehairi, E. | 2018 | Cross-sectional survey + chart review | United Kingdom |  |  |  |  |  |  |  |  |  | **✓** |  | Perceived versus actual prescribing practices may identify key areas for quality improvement. There were differences in the perceived and actual delirium assessment/plan and safety monitoring. |
| Boncyk, C.S. | 2021 | Retrospective cohort study | United States |  |  |  |  |  |  | **✓** | **✓** |  |  |  | Pharmacologic interventions, most often in the form of antipsychotic medications, for the treatment of ICU delirium are common, and often continued after delirium resolution and hospital discharge. |
| Brown, G. | 1998 | Quasi-experimental study | Canada |  |  |  |  |  |  |  |  | **✓** |  |  | With the implementation of a medication algorithm to promote sleep in the ICU with methotrimeprazine, there was no difference in the maximum number of continuous hours of sleep and additional sedating medications were required at night. |
| Ceraso, D.H. | 2010 | Cross-sectional survey | South America^‡^ | **✓** |  | **✓** |  |  |  |  |  |  |  |  | Despite considering delirium as a frequent, preventable problem and with serious repercussions for the critical patient, the intensivists surveyed did not use a tool for their evaluation or to guide antipsychotic medication prescribing in ICU. Efforts are necessary educational programs to disseminate the effectiveness and usefulness of the scales that early and accurate diagnosis of delirium in ICU. |
| Chawla, R. | 2013 | Cross-sectional survey | India | **✓** |  |  |  |  |  |  |  |  |  |  | Narcotics and non-narcotics are equally used analgesics. Haloperidol is the most common drug to treat delirium. Midazolam is the most commonly used sedative, but the current evidence driven use of fentanyl, propofol and dexmedetomidine is encouraging. |
| Collet, M.O. | 2019 | Focus groups | Denmark | **✓** |  |  |  |  |  |  |  |  |  |  | This study describes an algorithm of contemporary delirium management in Danish ICUs based on qualitative inquiry. When evidence-based solutions are unclear, nurses and physicians rely on personal experience, collective experience, and best available evidence to determine which patients to treat and what methods to use to treat ICU delirium. Delirium management still needs clear objectives and guidelines with evidence-based recommendations for first-line treatment and subsequent treatment options. |
| D’Angelo, R.G. | 2019 | Pre-post interventional study | United States |  | **✓** |  |  | **✓** |  |  |  |  |  |  | This is the first study to demonstrate a reduction in antipsychotic continuation at transition from the MICU after implementation of an antipsychotic discontinuation bundle in ICU patients. The authors believe this bundle allows for safer transitions of care from the MICU and decreases unnecessary antipsychotic therapy. |
| DeBacker, J. | 2018 | Retrospective cohort study | Canada |  | **✓** |  |  |  |  |  |  |  |  |  | Withdrawal from large doses of benzodiazepines and opioids administered over many days may play a role in the high incidence of delirium in patients on ECMO and warrants more investigation. If sedation minimization is achieved early after ECMO initiation, delirium and withdrawal syndromes may be reduced, thus allowing earlier and more aggressive mobilization. |
| Devlin, J.W. | 2011 | Cross-sectional survey | United States | **✓** |  |  |  |  |  |  |  |  |  |  | Practices and perceptions among the critical care pharmacists who responded to our survey regarding delirium recognition and treatment vary widely and are frequently not evidence-based. The survey findings may be related, in part, to the fact that there is a lack of rigorous evidence to guide many current ICU delirium recognition and treatment practices. While knowledge gaps surrounding ICU delirium recognition, prevention, and treatment among the pharmacists who responded to the survey are numerous, increased formal educational activities in this area should go a long way to improving pharmacists' knowledge and practice in this area. |
| Dyal, S. | 2019 | Cross-sectional survey | United States | **✓** |  |  |  |  |  |  |  |  |  |  | Assessment and management strategies of acute severe alcohol withdrawal vary considerably. Benzodiazepines are the mainstay of treatment. Atypical antipsychotics, haloperidol, among other sedative medications were all found to be significantly more likely to be utilized for prevention of central nervous system depression. |
| Dzierba, A.L. | 2019 | Cross-sectional survey | United States | **✓** |  | **✓** |  |  |  |  |  |  |  |  | Most respondents use validated scales and protocols to assess and manage pain, agitation/sedation, and delirium. The majority of respondents reported targeting a deep level of sedation with propofol being used for both deep and light levels of sedation. Reported delirium prevention strategies for patients on VV-ECMO include scheduled antipsychotics and scheduled haloperidol. Delirium treatment strategies include scheduled haloperidol and as needed atypical antipsychotics. |
| Eastwood, G.M. | 2012 | Quality improvement study | Australia |  | **✓** |  |  |  |  |  |  |  |  |  | Critical care nurses in one Australian ICU who responded to our survey think delirium assessment is important. Although they find unstructured assessments easier to perform, they wanted to persist with the CAM-ICU, in part because it facilitated more appropriate pharmacological treatment of delirium for their patients. Twice as much olanzapine and nearly five times as much haloperidol was prescribed in the CAM-ICU period. |
| Ely, E.W. | 2004 | Cross-sectional survey | United States | **✓** |  |  |  |  |  |  |  |  |  |  | The results of this survey provide data that show an overall appreciation for delirium as an important form of organ dysfunction yet point to a general disconnect between the perceived importance of delirium in the ICU and current practices of delirium monitoring and treatment. Common treatments for delirium reported by respondents include predominantly haloperidol followed by atypical antipsychotics. |
| Farrokh, S. | 2017 | Retrospective cohort study | United States |  | **✓** |  | **✓** |  |  |  |  |  |  |  | Atypical antipsychotics initiated in the ICU were frequently continued after hospital discharge in our institution. Given the known risks associated with extended therapy, initiatives are needed to prevent inappropriate continuation beyond hospitalization. |
| Flores, D.J. | 2015 | Cross-sectional survey | United States | **✓** |  | **✓** |  |  |  |  |  |  |  |  | Education improved staff understanding of the clinical implications of patients with delirium and treatment. |
| Gilani, A.A. | 2020 | Cross-sectional survey | United Kingdom | **✓** |  | **✓** |  |  |  |  |  |  |  |  | There is great amount of variation in the treatment of delirium that may represent clinical experience and familiarity with agents and assessments, and the dearth of positive research results. Typical and atypical antipsychotics were commonly prescribed for delirium. Most practitioners reported de-escalating treatment after delirium resolved by gradual weaning of treatment over time. |
| Gilbert, B. | 2017 | Retrospective cohort study | United States |  | **✓** |  | **✓** |  |  |  |  |  |  |  | Continuation of neuroleptics initiated for acute illness in the ICU upon discharge from the unit and hospital is highly prevalent in line with evidence currently published in the literature. The risk of being discharged on these agents does not appear to be any more prevalent based on admitting service, admission diagnosis, requirement of MV, or even selection of sedative utilized. Instead, continuation of neuroleptics upon transitions of care appeared to be more prevalent among patients receiving sleep aids, those with negative urine drug screen, and those requiring initiation of multiple neuroleptics during ICU stay. These data support the need for a hospital protocol identifying patient populations susceptible to the inappropriate continuation of neuroleptics and facilitating their discontinuation, avoiding unnecessary adverse events and costs. |
| Gill, K.V. | 2012 | Cross-sectional survey + retrospective cohort study | United States | **✓** | **✓** |  |  |  |  |  |  |  |  |  | Haloperidol was the most commonly prescribed antipsychotic overall, primarily because of its high use on an as-needed basis. Of the patients receiving a scheduled (not as-needed) antipsychotic, quetiapine was used most often. For all ICU types, haloperidol was selected as the preferred drug for delirium. Observed differences between perceived and actual sedation practices, as well as the limited use of protocols, raise important questions regarding the challenges of the overall management of sedation in the US. |
| Glass, M. | 2018 | Pre-post interventional study | Not reported |  |  |  |  |  |  | **✓** |  |  |  |  | Medication assessment by pharmacists in patients screening positive for ICU delirium resulted in less haloperidol use. |
| Gong, Z. | 2009 | Cross-sectional survey | China | **✓** |  | **✓** |  |  |  |  |  |  |  |  | Delirium was believed to be a significant or serious problem and under-diagnosis was acknowledged by respondents. When asked what drugs should be used to treat delirium, respondents mentioned haloperidol and olanzapine. However, in their clinical practice, few doctors used these drugs to deal with the patient's delirium specifically. The vast majority of respondents had little knowledge on the diagnosis and the standard treatment of delirium. |
| Johnson, K. | 2016 | Pre-post interventional study | United States | **✓** |  | **✓** |  |  |  |  |  |  |  |  | An educational intervention emphasising the importance of screening for delirium, risk factors for delirium and approaches to decrease the incidence of delirium can improve identifying and correctly treating delirium in a critical care setting. The two most frequent reasons cited for haloperidol pre-survey included minimal adverse side effects compared to lorazepam and less sedation. The two most frequent reasons cited for haloperidol post-questionnaire included less sedation and more effective than lorazepam with an increase in use of haloperidol. |
| Kim, D.H. | 2018 | Retrospective cohort study | United States |  | **✓** |  |  |  |  |  |  |  |  |  | In hospitalized older patients after cardiac surgery, we found that the rates of off-label antipsychotic medication use and potentially excessive dosing has declined, but substantial hospital-level variation and rapidly increasing trend in quetiapine use are concerning. To promote appropriate antipsychotic medication prescribing and improve clinical outcomes of older cardiac surgical patients, high-quality evidence on the effectiveness and harm of antipsychotics for management of delirium and training of health care providers about effective non-pharmacological interventions are urgently needed. |
| Kloet, M.A. | 2017 | Quality improvement study | United States |  |  |  |  |  | **✓** |  |  |  |  |  | In the ICU, antipsychotics were the most commonly encountered box warning drugs. These findings demonstrated the liberal use of antipsychotics that occurs in an ICU setting. Health care professionals should weigh the benefits of antipsychotic use against their risks, considering their questionable efficacy for ICU delirium |
| Kotfis, K. | 2017 | Cross-sectional survey | Poland | **✓** |  | **✓** |  |  |  |  |  |  |  |  | A majority of Polish ICUs do not adhere to international guidelines regarding sedation and delirium practices. High usage of benzodiazepines for sedation and ICU delirium treatment reveals persistence of non-evidence-based practice. Most frequently cited antipsychotic medications treat delirium were haloperidol following by atypical antipsychotics. |
| Kram, B.L. | 2015 | Retrospective cohort study | United States |  |  |  | **✓** |  |  |  |  |  |  |  | Atypical antipsychotic prescribing in the ICU is associated with significant patient-centered implications, despite limited data supporting long-term benefit when initiated in this setting. Providers should assess the indication for atypical antipsychotics initiated in the ICU routinely and discontinue these medications before ICU transfer and hospital discharge appropriately to avoid prolonged and possibly unnecessary use. |
| Kram, B.L. | 2019 | Quality improvement study | United States |  |  |  | **✓** | **✓** |  |  |  |  |  |  | Implementation of a pharmacy-initiated electronic handoff tool may reduce the proportion of atypical antipsychotic-naive ICU survivors with an atypical antipsychotic continued at the time of ICU transfer. The handoff tool was not associated with a significant reduction in the discharge prescribing rates of atypical antipsychotics for hospital survivors, but a clinically meaningful reduction was possibly achieved due to enhanced communication enabled by this tool. |
| Levine, A.R. | 2019 | Retrospective cohort study | United States |  |  |  |  | **✓** |  |  |  |  |  |  | A significant percentage of medical ICU and surgical ICU patients newly initiated on atypical antipsychotics remained on these agents at hospital discharge. Several risk factors influencing continuation of therapy existed in the two cohorts. Age ⩾60 years, pre-existing dementia, hemorrhagic stroke, and initiation of risperidone were associated with continuation of antipsychotics at discharge in the medical ICU cohort. In the surgical ICU cohort, patients with TBI and those initiated on quetiapine were more likely to be discharged on antipsychotics. The high percentage of older patients and those with dementia prescribed atypical antipsychotics at discharge is concerning, given the known risks associated with long-term use in these patients. Implementation of strategies to prevent delirium and actively wean off antipsychotics once delirium has resolved may help reduce initiation and unnecessary continuation of these agents at hospital discharge. |
| Mac Sweeney, R. | 2010 | Cross-sectional survey | United Kingdom | **✓** |  | **✓** |  |  |  |  |  |  |  |  | UK consultant intensivists seem to recognise the significance of delirium in critically ill patients but despite this screening with validated tools is uncommon and hypoactive delirium is rarely treated. Haloperidol is the most common agent chosen to treat both hyper- and hypo-active delirium, in spite of concerns about side effects in non-ICU populations. This survey was undertaken to provide information on usual care of delirium in critically ill patients in the UK. |
| Marshall, J. | 2016 | Retrospective cohort study | United States |  |  |  |  | **✓** |  |  |  |  |  |  | The authors found that newly initiated antipsychotic therapy is a common occurrence in the ICU and that approximately one fifth of newly initiated patients are discharged from the hospital with these medications newly added to their medication lists in this single-center study. Perhaps even more concerning, we have identified that a patient’s likelihood of continuing on these medications may not be entirely driven by the clinical needs of the patient but rather by nonclinical factors such as the type of antipsychotic used. Additional research is needed to better define the role of antipsychotic therapy post–critical illness and better delineate which patient populations would be suitable for continued treatment. |
| Mehta, S. | 2007 | Cross-sectional survey | Canada | **✓** |  |  |  |  |  |  |  |  |  |  | The results of this survey indicate that many ICU nurses are not content with current sedation and analgesia strategies and that most would welcome a strategy incorporating a protocol and a sedation scale. The most commonly reported antipsychotic used for sedation and analgesia in this survey was haloperidol. |
| Mo, Y. | 2017 | Cross-sectional survey | United States | **✓** |  | **✓** |  |  |  |  |  |  |  |  | This study demonstrates that ICU delirium practices have dramatically changed over the past decade. A majority of critical care practitioners were fully aware of the key components of the revised practice guidelines with regard to delirium management, such as delirium assessments and early mobilization. Respondents agreed that there is a need for well-conducted clinical trials to determine if haloperidol and atypical antipsychotics reduce the duration of delirium in adult ICU patients. |
| Palacios-Ceña, D. | 2016 | Focus groups | Spain | **✓** |  | **✓** |  |  |  |  |  |  |  |  | Our findings highlight how professionals perceive individuals with delirium. Doctors have difficulties selecting the appropriate drug, and for some patients, the dosage of the same is seen to vary across different shifts. Nurses believe that for the doctor, delirium is not a matter of urgency, and therefore the attention is often delayed. On the other hand, nurses have difficulty in applying verbal restraint, sleep management and early mobilisation, and there is a tendency towards the use of physical restraint while awaiting medical recommendations. The absence of a delirium protocol generates conflicts regarding which path of care to apply, especially during the night shift.  The complexity of delirium itself, together with the associated therapeutic variety, and the presence of difficulties in the application of care, can lead to inappropriate patient management. These results may  help to understand how doctors and nurses apply  decision-making processes regarding delirium management. This study contributes to the evidence base suggesting that delirium in the ICU presents obstacles to  management despite current existing guidelines. |
| Patel, M. | 2019 | Retrospective cohort study | Not reported |  | **✓** |  | **✓** |  |  |  |  |  |  |  | Pharmacologic sleep aids (including atypical antipsychotics) which are newly initiated in the ICU were commonly continued upon transfer out of the ICU. |
| Patel, R.P. | 2009 | Cross-sectional survey | United States | **✓** |  | **✓** |  |  |  |  |  |  |  |  | The results of this survey show discordance between the opinion that delirium is an important factor in patient outcome and the current practices in delirium monitoring and treatment. Although the number of people using a validated screening tool for delirium has increased significantly, this number is lower than expected. Most healthcare practitioners reported using a sedation protocol. Haloperidol was the most common medication reported for the treatment of delirium followed by atypical antipsychotics. |
| Ranzani, O.T. | 2014 | Quality improvement study | Brazil |  | **✓** | **✓** |  |  |  |  |  |  |  |  | The implementation of a light sedation policy is feasible in a group of nonteaching hospitals, and systematic monitoring of sedative consumption seems to be a simple and objective instrument for supporting the accomplishment of protocol on a large scale. The consumption of haloperidol showed no changes in the secular trend or postintervention; however, the consumption of haloperidol significantly increased immediately after intervention. |
| Rhoney, D.H. | 2003 | Cross-sectional survey | United States | **✓** |  |  |  |  |  |  |  |  |  |  | For sedative agents, morphine, lorazepam, haloperidol, and midazolam were used for longer than 72 hours by a majority of the respondents. 21% of the represented ICUs do not monitor sedation. |
| Salluh, J.I.F. | 2009 | Cross-sectional survey | Brazil | **✓** |  | **✓** |  |  |  |  |  |  |  |  | This survey provides valuable data on the perceived attitudes of Brazilian ICU physicians regarding sedation and delirium. Although delirium is acknowledged by most respondents as a severe medical condition, few systematic tools are used in clinical practice for the evaluation and treatment of delirium. Haloperidol and atypical antipsychotics were the most commonly reported antipsychotics used for delirium. The results of the present survey reemphasize the need to implement widespread educational efforts for the implementation of evidence-based strategies for the use of sedatives and the detection, monitoring, and treatment of delirium in ICU patients. |
| Selim, A.A. | 2017 | Cross-sectional survey | Egypt | **✓** |  | **✓** |  |  |  |  |  |  |  |  | Intensive care unit healthcare professionals do not have adequate training or routine screening of delirium. There is an evident absence of using standardised tools or adapting protocols to monitor and manage delirium. First-line treatment choices for delirium included sedatives and haloperidol. |
| Silverman, D. | 2013 | Retrospective cohort study | United States |  | **✓** | **✓** |  |  |  |  |  |  |  |  | Quetiapine administration may reduce benzodiazepine requirements and duration of restraint use. There is a high prevalence of continuation of quetiapine beyond the intensive care unit environment. This is a potential area for quality improvement with regards to medication reconciliation, limiting adverse effects, drug interactions, and cost. QTc interval should be monitored in patients receiving quetiapine. |
| Stuart, M.M. | 2020 | Quasi-experimental study (retrospective) | United States |  | **✓** |  | **✓** | **✓** |  |  |  |  |  |  | The implementation of a protocol for pharmacists with prescriptive authority to discontinue antipsychotics initiated for ICU delirium once ICU delirium resolves significantly decreases the rate of antipsychotic continuation at hospital discharge without increasing the recurrence of ICU delirium or QTc prolongation. This study demonstrated the impact of pharmacists assisting physicians in determining when antipsychotics can be discontinued to safely stop the medication prior to hospital discharge. Future studies are needed to assess antipsychotic discontinuation in the ICU setting, evaluate the need for tapering of antipsychotics, and determine the safety and efficacy of shorter duration taper protocols. |
| Swan, J.T. | 2012 | Retrospective cohort study | United States |  | **✓** |  |  |  |  |  |  |  |  |  | Antipsychotics are administered to 1 in every 10 ICU patients, and exposure to these medications is associated with increased ICU and hospital length of stay. Patients exposed to an antipsychotic, when there is no documentation of a mental disorder, have increased ICU length of stay, hospital length of stay and mortality compared to patients with documentation of a mental disorder. These findings do not support the use of antipsychotic medications in the ICU when patients do not have a documented diagnosis of a mental disorder or delirium. The appropriate indication and agent selection of the antipsychotics should continue to be studied in prospective, randomized, controlled trials. Due to the high prevalence of antipsychotic use in ICU patients who do not have a documented mental disorder, future studies are needed to describe the specific indications for antipsychotics and common doses that are being used in critically ill patients in current clinical practice. |
| Sztrymf, B. | 2012 | Cross-sectional survey | France | **✓** |  | **✓** |  |  |  |  |  |  |  |  | This study reports the findings of a French national survey on delirium screening and its management. Even though French ICU physicians participating in our survey are aware of the possible severity of delirium, they rarely use a dedicated and validated screening tool. Early patient mobilization is less frequent than declared as possible, mainly in mechanically ventilated patients. Treatments for delirium are reported to include antipsychotics, benzodiazepines, and hydroxyzine. |
| Thiboutot, Z. | 2016 | Prospective cohort study | Canada |  | **✓** |  |  |  |  |  |  |  |  |  | Delirium is increasingly associated with negative clinical outcomes, and recent guidelines have highlighted the importance of appropriate screening and modification of risk factors. This multicentre study identified infrequent use of delirium screening tools in Canadian ICUs. Antipsychotics were prescribed frequently, and patterns of use were variable. There is an opportunity to improve delirium screening and management of mechanically ventilated patients in Canadian ICUs. |
| Tomichek, J.E. | 2016 | Prospective cohort study | United States |  | **✓** |  | **✓** |  |  |  |  |  |  |  | In a large cohort of patients recovering from critical illness, antipsychotics were prescribed at hospital discharge to one out of every four patients newly treated with antipsychotics for delirium in the ICU, a practice most likely to occur among patients treated with an atypical antipsychotic in the hospital. Not only are the efficacy and safety of antipsychotics for delirium in the ICU unproven, but it remains unclear which antipsychotic, if any, should be used to treat delirium and for how long. Until clear evidence from large randomized trials is available regarding the efficacy and appropriate duration of antipsychotic use for delirium in the ICU, this class of medication should be used with caution. Focused efforts should be implemented to ensure antipsychotics are appropriately discontinued upon transitions of care in the hospital. |
| Trogrlic, Z. | 2013 | Cross-sectional survey | Netherlands | **✓** |  | **✓** |  |  |  |  |  |  |  |  | Our survey showed that healthcare professionals considered delirium an important but underdiagnosed form of organ failure. In contrast, screening tools for delirium are scarcely used, knowledge can be improved and protocolled treatment based on positive screening is often lacking. Haloperidol was the first-choice pharmacologic agents for the treatment of delirium. These results suggest that the focus of implementation of ICU delirium management should not be on motivational aspects, but on knowledge improvements, training in screening tools and implementation of treatment and prevention protocols. |
| Trogrlic, Z. | 2013 | Prospective cohort study | Netherlands |  | **✓** | **✓** |  |  |  |  |  |  |  |  | Daily screening for ICU delirium with a validated screening instrument is applied in less than one-half of the time in critically ill patients and management of delirium is often not guided by this screening. Haloperidol was used as the first-choice medication. Measures aimed at delirium prevention were carried only in a small minority. To implement protocolled delirium care in the region at study, a multifaceted tailored implementation program is needed. |
| van den Boogaard, M. | 2009 | Quality improvement study | Netherlands |  | **✓** |  |  |  |  |  |  |  |  |  | Tailoring an implementation strategy to the needs of the ICU was successful. The main goals were achieved within a relatively short time. Early recognition of delirium with the CAM-ICU has become a standard component of daily care by the nurses in the ICU and contributes to the quality of care. Early detection of delirium leads to lower dosage and shorter periods of haloperidol treatment in critically ill patients. |
| Wang, J. | 2017 | Cross-sectional survey | China | **✓** |  | **✓** |  |  |  |  |  |  |  |  | The practice of pain, agitation, and delirium assessment and management in China was in accordance with the international situations. The guideline and the updated recommendations were accepted by most of the clinicians in China. Haloperidol second most common medication used for delirium after dexmedetomidine. |
| **Inpatient** | | | | | | | | | | | | | | | |
| Al-Qadheeb, N.S. | 2013 | Prospective cohort study | United States |  | **✓** |  |  |  |  |  |  |  |  |  | Among long-term acute care hospital patients requiring permanent mechanical ventilation, scheduled antipsychotic therapy is used frequently and is associated with a greater incidence of psychiatric evaluation, delirium, as-needed antipsychotic use, and sitter use. Although scheduled antipsychotic therapy was used, related adverse effects are uncommon, and these effects are infrequently monitored |
| Basciotta, M. | 2018 | Retrospective cohort study | United States |  |  |  |  |  |  |  | **✓** |  |  |  | In hospitalized adults, typical antipsychotics may be associated with increased mortality or cardiopulmonary arrest, while atypical antipsychotics may only be associated with increased risk among adults age 65 and older. |
| Bascom, P.B. | 2014 | Case report or case series | USA |  |  |  |  |  |  |  |  | **✓** |  |  | Agitated delirium is a palliative care emergency. High doses of neuroleptic medications, with rotation to an alternate neuroleptic when side effects occur with standard haloperidol, may effectively palliate agitated delirium. |
| Bedouch, P. | 2015 | Cross-sectional survey | France | **✓** |  |  |  |  |  |  |  |  |  |  | The results of this large evaluation study of multiple medications including typical and atypical antipsychotics show that only a few types of drugs and errors constitute a substantial proportion of daily routine pharmacists’ interventions. Various predictors of physicians’ acceptance of pharmacist interventions are identified such as drug groups, intervention type, ward specialty and the degree of pharmacist on ward integration. |
| Birigen, E.K. | 2021 | Cross-sectional survey | United States | **✓** |  |  |  |  |  |  |  |  |  |  | Survey respondents indicated that formal education, medical literature, and input from palliative care and pharmacology colleagues informed their antipsychotic prescribing practices; a formal care pathway should integrate these different sources of information and provide specific resources in the community to help oncology providers connect their patients to long-term specialized psychiatric and therapeutic care. |
| Brennan, M. | 2018 | Case-control | USA |  | **✓** |  |  |  |  |  |  |  |  |  | Lower acute care of the elder (ACE) unit use of new antipsychotics may reflect better socialization, lower delirium rates, improved prescribing, early mobilization, skilled staff or an adjusted environment. The ACE team may have managed/prevented milder cases of agitation so that patients receiving antipsychotics were more distressed than their non-ACE peers. |
| Brett, J. | 2020 | Retrospective cohort study + chart review | Australia |  | **✓** |  |  |  |  |  |  |  |  |  | Off-label prescribing of quetiapine was common in this sample of inpatients, and senior hospital staff should remain cautious of quetiapine prescribing for indications where the evidence of harms and benefits remains unclear. Communication with community prescribers could also be improved to reduce the risk of conversion from intended short-term off-label use to longer-term use. |
| Briskman, I. | 2010 | Retrospective cohort study | Israel |  | **✓** |  |  |  |  |  |  |  |  |  | Risperidone may be the drug of choice for the treatment of delirium. However, due to the limitations inherent in a retrospective analysis and other methodological limitations, prospective large-scale trials are needed to support this recommendation. |
| Costa-Dias, M.J. | 2014 | Retrospective cohort study | Portugal |  |  |  |  |  |  |  |  |  |  | **✓** | Of the participants who took antipsychotic drugs, there was a seven times increased odds risk of fall and five times more risk of recurrent falls. The most common drug prescribed was haloperidol, |
| Fontaine, G.V. | 2018 | Retrospective cohort study | United States |  | **✓** |  | **✓** |  |  |  |  |  |  |  | Antipsychotics may be inappropriately continued in non-psychiatric patients at hospital discharge. Strategies to limit the number of potentially inappropriate antipsychotic prescriptions at hospital discharge should be evaluated to reduce the undue adverse effect burden and emergency department visits associated with antipsychotic use. |
| Herzig, S.J. | 2016 | Retrospective cohort study | United States |  | **✓** |  |  |  |  |  |  |  |  |  | in this large cohort of nonpsychiatric admissions to 300 US hospitals, antipsychotic medication exposure was common, often at high daily doses. Delirium and dementia were the strongest predictors of use among the patient and hospital characteristics examined. The variation in antipsychotic prescribing was not fully accounted for by measured patient characteristics and raises the possibility of differing hospital prescribing cultures. |
| Hosie, A. | 2021 | Cross-sectional survey | Australia (All) | **✓** |  |  |  |  |  |  |  |  |  |  | Clinicians’ use of antipsychotic during delirium remains common and is primarily motivated by distress and safety concerns for the patient and others nearby. Delirium-related distress and safety concerns for patients, family, staff and others nearby are clinically meaningful and should be explicitly acknowledged and addressed in healthcare institutions and future studies. |
| Hui, D. | 2011 | Retrospective cohort study | United States |  | **✓** |  |  |  |  |  |  |  |  |  | In unselected advanced cancer patients with delirium in an acute palliative care unit, the median daily dose of neuroleptics was low, raising questions regarding the effectiveness of current neuroleptic use by palliative care specialists for management of delirium. Findings from this study highlight the need for prospective clinical trials to determine the safety, optimal dose, titration strategy and most appropriate combinations of neuroleptics for effective management of delirium symptoms, delirium recall and related distress. |
| Kuscu, M.K. | 2004 | Cross sectional survey + semi-structured interviews | Turkey | **✓** |  |  |  |  |  |  |  |  |  |  | Delirium remains an important clinical emergency in clinical practice. The evaluation of the attitudes of resident physicians toward delirium management including antipsychotic medication use will provide ground to develop new consensus guidelines for management of delirium. |
| Loh, E.C. | 2011 | Case report or case series | Malaysia |  |  |  |  |  |  | **✓** |  |  |  |  | Orodispersible olanzapine when used judiciously may have a useful role in managing refractory terminal delirium in palliative care setting and merits further study. |
| Loh, K.P. | 2016 | Retrospective cohort study + chart review | United States |  | **✓** |  | **✓** |  |  |  |  |  |  |  | Initiating an antipsychotic in the hospital is likely to result in long-term use of these medications despite associated antipsychotic risk of falls, fractures, stroke, cardiovascular disease, and increased mortality in those with underlying dementia. When possible, behavioral interventions to prevent delirium and slow the trajectory of decline should be implemented to reduce antipsychotic use. |
| Masman, A.D. | 2015 | Retrospective cohort study | Netherlands |  | **✓** |  |  |  |  |  |  |  |  |  | Haloperidol was commonly prescribed in combination with morphine and/or midazolam on day of death. |
| Mattison, M.L.P. | 2014 | Pre-post control interventional study | United States |  | **✓** |  |  |  |  |  |  |  |  |  | An intervention focused on delirium prevention and recognition by bedside staff combined with computerized decision support facilitates safer prescribing of high risk medications including antipsychotics, may results in less need for extended care. |
| McNeill, R. | 2021 | Retrospective cohort study | New Zealand |  |  |  |  |  | **✓** |  |  |  |  |  | This study compared the two deprescribing tools OncPal and STOPPFrail to an expert clinical review in an unselected palliative population. Haloperidol was the most common false positive drug identified for STOPPFrail. |
| Meagher, D. | 2013 | Cross-sectional survey | Europe^†^ | **✓** |  | **✓** |  |  |  |  |  |  |  |  | Delirium awareness, delirium knowledge, and lack of education cited as most commonly reported barriers to improving the detection of delirium. Non-pharmacologic interventions were the first choice in hypoactive delirium and a combination of non-pharmacologic and pharmacologic interventions (using haloperidol and risperidone) were the first-line choice for delirium management. |
| Someya, T. | 2001 | Cross-sectional cohort study | Japan | **✓** |  |  |  |  |  |  |  |  |  |  | The present study discusses the results of a study on the use of medications for delirium, a condition commonly found in inpatients of general hospitals in Japan. Haloperidol was perceived as the first-line choice for the treatment of delirium. Due to fewer perceived side effects. |
| Thacker, S. | 1996 | Cross-sectional survey | United Kingdom | **✓** |  |  |  |  |  |  |  |  |  |  | This survey examines the prescribing opinions of junior doctors likely to be required to rapidly sedate an acutely agitated elderly patient. This study highlights the need for junior doctors to receive continuing education on the use of psychotropic drugs. |
| Trenaman, S.C. | 2018 | Cross-sectional cohort study | Canada |  | **✓** |  |  |  |  |  |  |  |  |  | There were no risk factors identified to predict continuation of an antipsychotic after a fall-related hospitalization. Prescribing quality may be assessed on a population level. |
| Tropea, J. | 2009 | Medical record audit | Australia |  | **✓** |  |  |  |  |  |  |  |  |  | This study provides valuable baseline information about what areas of practice are consistent with the guideline recommendations for the management of older people with severe symptoms of delirium and highlights which areas should be the focus for future quality improvement. In particular, commencing the antipsychotic agent at a low dose; and documentation of a clear management plan appear to be vital. |
| Weir, D.L. | 2020 | Prospective cohort study | Canada |  | **✓** |  | **✓** |  | **✓** |  |  |  |  |  | The incidence of potentially inappropriate medication prescribing (including antipsychotic medication prescribing) attributed to hospitalization is high, and this is associated with an increase in adverse drug events, emergency department visits, rehospitalizations, and death within 30 days of discharge. |
| Wong, A. | 2014 | Retrospective cohort study | Canada |  | **✓** |  |  |  |  |  |  |  |  |  | In the acute setting, psychiatrists and geriatricians may prescribe intramuscular olanzapine for behavioural symptoms in elderly patients. Commonly experienced adverse effects include lethargy, drowsiness, and constipation. Close monitoring is the key to ensuring safe use. |
| Yasuyuki, O. | 2016 | Cross-sectional survey | Japan | **✓** |  |  |  |  |  |  |  |  |  |  | There were areas of agreement and a lack of consensus regarding the first-line pharmacological treatment for delirium with a diverse range of clinical features. In the absence of a definitive treatment trial, most experts preferred to use risperidone or quetiapine for hyperactive delirium. These results highlight a need for a high-quality placebo-controlled trial to allow a definitive conclusion to be reached on the efficacy of risperidone and quetiapine for hyperactive delirium. |
| **Emergency department** | | | | | | | | | | | | | | | |
| Bervoets, C. | 2015 | Cross-sectional survey | Belgium | **✓** |  |  |  |  |  |  |  |  |  |  | There is no clear or systematic rationale for prescribing for acute agitation in Belgium. Practice in treating acute agitation shows a complex relationship with published evidence and guidelines. The level of agitation in patients and the type of physician prescribing the first pharmaceutical treatment both are clearly important variables and should be implemented in further research designs. |
| Campillo, A. | 2012 | Retrospective cohort study | United States |  |  |  |  |  |  |  |  | **✓** |  |  | There appeared to be limited effects on vital signs in this small sample when using a combination of haloperidol and lorazepam. |
| Chan, E.W. | 2015 | Cross-sectional survey | Hong Kong | **✓** |  |  |  |  |  |  |  |  |  |  | The use of haloperidol and benzodiazepines as monotherapy is common in the management of acute agitation in Hong Kong emergency departments. Prescribers’ choice of sedation drugs are also more conservative and less variable overall. Future work could focus on clinical practice guideline development and training regarding the safe use of combination therapy. |
| Chan, E.W. | 2011 | Cross-sectional survey | Australia | **✓** |  |  |  |  |  |  |  |  |  |  | There is considerable variation in the management of hypothetical cases of acute agitation in Australasian emergency departments. Benzodiazepines and antipsychotics, either alone or in combination, are commonly used. An Australasian clinical practice guideline was perceived as useful. |
| Cowling, M. | 2019 | Cross-sectional survey | United States | **✓** |  |  |  |  |  |  |  |  |  |  | Emergency department providers reported using haloperidol most often as a second line treatment to manage both acute and acute on chronic pain. When haloperidol was used as a first line agent, providers claimed that additional medicines were not usually required. Haloperidol may provide an effective alternative to opioids in treatment of acute pain and acute exacerbations of chronic pain in the emergency department. |

*As reported in the study

**Supplementary Table 4.** Reported antipsychotic medication prescribing indications

included studies by acute care setting

| REPORTED ANTIPSYCHOTIC PRESCRIBING INDICATIONS | ACUTE CARE SETTING | | |
| --- | --- | --- | --- |
|  | **Intensive care^1^**  N=43 | **Inpatient^1^**  N=21 | **Emergency department**  N=5 |
| Delirium | 34 (79%) | 14 (67%) | 0 (0%) |
| Agitation | 9 (21%) | 7 (33%) | 4 (80%) |
| Sedation | 5 (12%) | 1 (5%) | 0 (0%) |
| Sleep | 5 (12%) | 2 (10%) | 0 (0%) |
| Palliation | 0 (0%) | 4 (19%) | 0 (0%) |
| Alcohol withdrawal | 1 (2%) | 0 (0%) | 0 (0%) |
| Pain | 1 (2%) | 1 (5%) | 1 (20%) |
| Antiemetic | 0 (0%) | 2 (10%) | 0 (0%) |
| Dementia | 0 (0%) | 1 (5%) | 0 (0%) |
| Anxiety/panic | 0 (0%) | 1 (5%) | 0 (0%) |
| Reduce falls | 0 (0%) | 1 (5%) | 0 (0%) |

Percentages do not add up to 100 due to the possibility of multiple outcomes per study

^1^One primary study report outcomes for patients admitted as inpatients and in ICU

**Supplementary Table 5.** Measured and perceived antipsychotics prescribed and prescribing indications reported for included studies, by acute care setting

| **Intensive care** | | | | | | | | | | | | | | | | |
| --- | --- | --- | --- | --- | --- | --- | --- | --- | --- | --- | --- | --- | --- | --- | --- | --- |
| **First author** | **Year** | **Study type** | **Country/Continent** | **Prescribed antipsychotic medications** | | **Antipsychotic indication** | | | | | | | | | | |
|  |  |  |  | **Perceived** | **Measured** | **Delirium** | **Agitation** | **Sedation** | **Sleep** | **Palliation** | **Alcohol withdrawal** | **Dementia** | **Antiemetic** | **Anxiety/**  **Panic** | **Reduce falls** | **Pain** |
| Almehairi, E. | 2018 | Cross-sectional survey + chart review | United Kingdom |  | Haloperidol  Quetiapine  Olanzapine | **✓** | **✓** |  |  |  |  |  |  |  |  |  |
| Boncyk, C.S. | 2021 | Retrospective cohort study | United States |  | Haloperidol  Quetiapine  Olanzapine | **✓** |  |  |  |  |  |  |  |  |  |  |
| Brown, G. | 1998 | Quasi-experimental study | Canada |  | Methotrimeprazine |  |  |  | **✓** |  |  |  |  |  |  |  |
| Ceraso, D.H. | 2010 | Cross-sectional survey | South America^‡^ | Haloperidol |  | **✓** |  |  |  |  |  |  |  |  |  |  |
| Chawla, R. | 2013 | Cross-sectional survey | India | Haloperidol |  | **✓** |  |  |  |  |  |  |  |  |  |  |
| Collet, M.O. | 2019 | Focus groups | Denmark | Haloperidol  Olanzapine |  | **✓** | **✓** |  |  |  |  |  |  |  |  |  |
| D’Angelo, R.G. | 2019 | Pre-post interventional study | United States |  | Haloperidol  Quetiapine  Olanzapine  Risperidone | **✓** |  |  |  |  |  |  |  |  |  |  |
| DeBacker, J. | 2018 | Retrospective cohort study | Canada |  | Haloperidol  Quetiapine | **✓** |  |  |  |  |  |  |  |  |  |  |
| Devlin, J.W. | 2011 | Cross-sectional survey | United States | Haloperidol  Quetiapine  Olanzapine |  | **✓** | **✓** |  |  |  |  |  |  |  |  |  |
| Dyal, S. | 2019 | Cross-sectional survey | United States | Haloperidol |  |  |  |  |  |  | **✓** |  |  |  |  |  |
| Dzierba, A.L. | 2019 | Cross-sectional survey | United States | Haloperidol |  | **✓** |  |  |  |  |  |  |  |  |  |  |
| Eastwood, G.M. | 2012 | Quality improvement study | Australia |  | Haloperidol  Quetiapine  Olanzapine | **✓** |  |  |  |  |  |  |  |  |  |  |
| Ely, E.W. | 2004 | Cross-sectional survey | United States | Haloperidol  Quetiapine  Olanzapine  Risperidone |  | **✓** |  |  |  |  |  |  |  |  |  |  |
| Farrokh, S. | 2017 | Retrospective cohort study | United States |  | Quetiapine  Olanzapine  Risperidone  Aripiprazole  Ziprasidone | Not reported |  |  |  |  |  |  |  |  |  |  |
| Flores, D.J. | 2015 | Cross-sectional survey | United States | Haloperidol |  | **✓** |  |  |  |  |  |  |  |  |  |  |
| Gilani, A.A. | 2020 | Cross-sectional survey | United Kingdom | Haloperidol  Quetiapine  Olanzapine  Risperidone  Aripiprazole  Ziprasidone |  | **✓** |  |  |  |  |  |  |  |  |  |  |
| Gilbert, B. | 2017 | Retrospective cohort study | United States |  | Haloperidol  Quetiapine  Olanzapine  Risperidone | Not reported |  |  |  |  |  |  |  |  |  |  |
| Gill, K.V. | 2012 | Cross-sectional survey + retrospective cohort study | United States | Haloperidol  Quetiapine  Olanzapine  Risperidone | Haloperidol  Quetiapine  Olanzapine  Risperidone |  |  | **✓** |  |  |  |  |  |  |  |  |
| Glass, M. | 2018 | Pre-post interventional study | Not reported |  | Haloperidol | **✓** |  |  |  |  |  |  |  |  |  |  |
| Gong, Z. | 2009 | Cross-sectional survey | China | Haloperidol  Olanzapine |  | **✓** |  |  |  |  |  |  |  |  |  |  |
| Johnson, K. | 2016 | Pre-post interventional study | United States | Haloperidol |  | **✓** |  |  |  |  |  |  |  |  |  |  |
| Kim, D.H. | 2018 | Retrospective cohort study | United States |  | Haloperidol  Quetiapine  Olanzapine  Risperidone  Aripiprazole  Ziprasidone | Not reported |  |  |  |  |  |  |  |  |  |  |
| Kloet, M.A. | 2017 | Quality improvement study | United States |  | Risperidone | Not reported |  |  |  |  |  |  |  |  |  |  |
| Kotfis, K. | 2017 | Cross-sectional survey | Poland | Haloperidol  Quetiapine  Olanzapine |  | **✓** |  |  |  |  |  |  |  |  |  |  |
| Kram, B.L. | 2015 | Retrospective cohort study | United States |  | Quetiapine  Olanzapine  Risperidone  Aripiprazole  Ziprasidone | **✓** |  |  |  |  |  |  |  |  |  |  |
| Kram, B.L. | 2019 | Quality improvement study | United States |  | Quetiapine  Olanzapine  Risperidone  Aripiprazole | **✓** | **✓** | **✓** | **✓** |  |  |  |  |  |  |  |
| Levine, A.R. | 2019 | Retrospective cohort study | United States |  | Quetiapine  Olanzapine  Risperidone | **✓** |  |  | **✓** |  |  |  |  |  |  |  |
| Mac Sweeney, R. | 2010 | Cross-sectional survey | United Kingdom | Haloperidol |  | **✓** |  |  |  |  |  |  |  |  |  |  |
| Marshall, J. | 2016 | Retrospective cohort study | United States |  | Haloperidol  Quetiapine  Olanzapine  Risperidone  Aripiprazole  Ziprasidone | Not reported |  |  |  |  |  |  |  |  |  |  |
| Mehta, S. | 2007 | Cross-sectional survey | Canada | Haloperidol |  |  |  |  | **✓** |  |  |  |  |  |  |  |
| Mo, Y. | 2017 | Cross-sectional survey | United States | Haloperidol  Quetiapine |  | **✓** | **✓** |  |  |  |  |  |  |  |  |  |
| Palacios-Ceña, D. | 2016 | Focus groups | Spain | Haloperidol |  | **✓** |  |  |  |  |  |  |  |  |  |  |
| Patel, M. | 2019 | Retrospective cohort study | Not reported |  | Quetiapine |  |  |  | **✓** |  |  |  |  |  |  |  |
| Patel, R.P. | 2009 | Cross-sectional survey | United States | Haloperidol  Quetiapine  Olanzapine  Risperidone  Aripiprazole  Ziprasidone |  | **✓** |  |  |  |  |  |  |  |  |  |  |
| Ranzani, O.T. | 2014 | Quality improvement study | Brazil |  | Haloperidol |  |  | **✓** |  |  |  |  |  |  |  |  |
| Rhoney, D.H. | 2003 | Cross-sectional survey | United States | Haloperidol |  |  | **✓** | **✓** |  |  |  |  |  |  |  | **✓** |
| Salluh, J.I.F. | 2009 | Cross-sectional survey | Brazil | Haloperidol |  | **✓** |  |  |  |  |  |  |  |  |  |  |
| Selim, A.A. | 2017 | Cross-sectional survey | Egypt | Haloperidol |  | **✓** |  |  |  |  |  |  |  |  |  |  |
| Silverman, D. | 2013 | Retrospective cohort study | United States |  | Quetiapine |  | **✓** |  |  |  |  |  |  |  |  |  |
| Stuart, M.M. | 2020 | Quasi-experimental study (retrospective) | United States |  | Haloperidol  Quetiapine  Olanzapine  Risperidone  Ziprasidone | **✓** | **✓** |  |  |  |  |  |  |  |  |  |
| Swan, J.T. | 2012 | Retrospective cohort study | United States |  | Haloperidol  Quetiapine  Olanzapine  Risperidone  Ziprasidone | **✓** |  |  |  |  |  |  |  |  |  |  |
| Sztrymf, B. | 2012 | Cross-sectional survey | France | Haloperidol |  | **✓** |  |  |  |  |  |  |  |  |  |  |
| Thiboutot, Z. | 2016 | Prospective cohort study | Canada |  | Haloperidol  Quetiapine  Olanzapine  Risperidone |  | **✓** |  | **✓** |  |  |  |  |  |  |  |
| Tomichek, J.E. | 2016 | Prospective cohort study | United States |  | Haloperidol  Quetiapine  Olanzapine  Risperidone  Ziprasidone | **✓** |  |  |  |  |  |  |  |  |  |  |
| Trogrlic, Z. | 2013 | Cross-sectional survey | Netherlands | Haloperidol |  | **✓** |  |  |  |  |  |  |  |  |  |  |
| Trogrlic, Z. | 2013 | Prospective cohort study | Netherlands |  | Haloperidol | **✓** |  |  |  |  |  |  |  |  |  |  |
| van den Boogaard, M. | 2009 | Quality improvement study | Netherlands |  | Haloperidol | **✓** |  |  |  |  |  |  |  |  |  |  |
| Wang, J. | 2017 | Cross-sectional survey | China | Haloperidol |  | **✓** |  |  |  |  |  |  |  |  |  |  |
| **Inpatient** | | | | | | | | | | | | | | | | |
| Al-Qadheeb, N.S. | 2013 | Prospective cohort study | United States |  | Haloperidol  Quetiapine  Olanzapine  Risperidone  Aripiprazole  Ziprasidone | Not reported |  |  |  |  |  |  |  |  |  |  |
| Basciotta, M. | 2018 | Retrospective cohort study | United States |  | Haloperidol  Quetiapine  Olanzapine  Risperidone  Aripiprazole  Ziprasidone | Not reported |  |  |  |  |  |  |  |  |  |  |
| Bascom, P.B. | 2014 | Case report or case series | USA | Haloperidol  Quetiapine  Olanzapine  Risperidone |  | **✓** | **✓** |  |  | **✓** |  |  |  |  |  |  |
| Bedouch, P. | 2015 | Cross-sectional survey | France | Haloperidol |  | Not reported |  |  |  |  |  |  |  |  |  |  |
| Birigen, E.K. | 2021 | Cross-sectional survey | United States | Haloperidol  Quetiapine  Olanzapine |  |  |  |  | **✓** |  |  |  | **✓** | **✓** |  | **✓** |
| Brennan, M. | 2018 | Case-control | USA |  | Haloperidol  Quetiapine  Olanzapine  Risperidone | **✓** |  |  |  |  |  |  |  |  | **✓** |  |
| Brett, J. | 2020 | Retrospective cohort study + chart review | Australia |  | Quetiapine | **✓** | **✓** |  |  |  |  |  |  |  |  |  |
| Briskman, I. | 2010 | Retrospective cohort study | Israel |  | Haloperidol  Risperidone | **✓** |  |  |  |  |  |  |  |  |  |  |
| Costa-Dias, M.J. | 2014 | Retrospective cohort study | Portugal |  | Haloperidol | Not reported |  |  |  |  |  |  |  |  |  |  |
| Fontaine, G.V. | 2018 | Retrospective cohort study | United States |  | Haloperidol  Quetiapine  Olanzapine  Risperidone  Ziprasidone | Not reported |  |  |  |  |  |  |  |  |  |  |
| Herzig, S.J. | 2016 | Retrospective cohort study | United States |  | Haloperidol  Quetiapine  Olanzapine  Risperidone  Aripiprazole  Ziprasidone | **✓** |  |  |  |  |  | **✓** |  |  |  |  |
| Hosie, A. | 2021 | Cross-sectional survey | Australia (All) | Haloperidol  Quetiapine  Olanzapine  Risperidone |  | **✓** |  |  |  | **✓** |  |  |  |  |  |  |
| Hui, D. | 2011 | Retrospective cohort study | United States |  | Haloperidol  Olanzapine | **✓** |  | **✓** |  |  |  |  |  |  |  |  |
| Kuscu, M.K. | 2004 | Cross sectional survey + semi-structured interviews | Turkey | Haloperidol  Olanzapine  Risperidone |  | **✓** |  |  |  |  |  |  |  |  |  |  |
| Loh, E.C. | 2011 | Case report or case series | Malaysia | Olanzapine |  |  | **✓** |  |  | **✓** |  |  |  |  |  |  |
| Loh, K.P. | 2016 | Retrospective cohort study + chart review | United States |  | Haloperidol  Quetiapine  Olanzapine  Risperidone | **✓** |  |  |  |  |  |  |  |  |  |  |
| Masman, A.D. | 2015 | Retrospective cohort study | Netherlands |  | Haloperidol |  |  |  |  | **✓** |  |  |  |  |  |  |
| Mattison, M.L.P. | 2014 | Pre-post control interventional study | United States |  | Haloperidol |  | **✓** |  |  |  |  |  |  |  |  |  |
| McNeill, R. | 2021 | Retrospective cohort study | New Zealand |  | Haloperidol |  |  |  |  |  |  |  | **✓** |  |  |  |
| Meagher, D. | 2013 | Cross-sectional survey | Europe^†^ | Haloperidol  Risperidone |  | **✓** |  |  |  |  |  |  |  |  |  |  |
| Someya, T. | 2001 | Cross-sectional cohort study | Japan | Haloperidol |  | **✓** | **✓** |  |  |  |  |  |  |  |  |  |
| Thacker, S. | 1996 | Cross-sectional survey | United Kingdom | Haloperidol |  |  | **✓** |  |  |  |  |  |  |  |  |  |
| Trenaman, S.C. | 2018 | Cross-sectional cohort study | Canada |  | Haloperidol  Quetiapine  Olanzapine  Risperidone  Methotrimeprazine | Not reported |  |  |  |  |  |  |  |  |  |  |
| Tropea, J. | 2009 | Medical record audit | Australia |  | Haloperidol  Quetiapine  Olanzapine  Risperidone | **✓** |  |  |  |  |  |  |  |  |  |  |
| Weir, D.L. | 2020 | Prospective cohort study | Canada |  | Quetiapine  Olanzapine  Risperidone  Aripiprazole | **✓** |  |  |  |  |  |  |  |  |  |  |
| Wong, A. | 2014 | Retrospective cohort study | Canada |  | Olanzapine |  | **✓** |  |  |  |  |  |  |  |  |  |
| Yasuyuki, O. | 2016 | Cross-sectional survey | Japan | Haloperidol  Olanzapine  Risperidone  Aripiprazole  Methotrimeprazine |  | **✓** |  |  |  |  |  |  |  |  |  |  |
| **Emergency department** | | | | | | | | | | | | | | | | |
| Bervoets, C. | 2015 | Cross-sectional survey | Belgium | Haloperidol  Quetiapine  Olanzapine  Risperidone  Aripiprazole |  |  | **✓** |  |  |  |  |  |  |  |  |  |
| Campillo, A. | 2012 | Retrospective cohort study | United States |  | Haloperidol |  | **✓** |  |  |  |  |  |  |  |  |  |
| Chan, E.W. | 2015 | Cross-sectional survey | Hong Kong | Haloperidol |  |  | **✓** |  |  |  |  |  |  |  |  |  |
| Chan, E.W. | 2011 | Cross-sectional survey | Australia | Haloperidol |  |  | **✓** |  |  |  |  |  |  |  |  |  |
| Cowling, M. | 2019 | Cross-sectional survey | United States | Haloperidol |  |  |  |  |  |  |  |  |  |  |  | **✓** |

**Supplementary Table 6.** Number of studies reporting on healthcare professional reported perceived antipsychotic prescribing practices in acute care, by acute care setting and antipsychotic type

| **ACUTE CARE SETTING** |  | **PERCEIVED ANTIPSYCHOTIC PRESCRIBING PRACTICES**  N= 36 | | | | | |  |  |
| --- | --- | --- | --- | --- | --- | --- | --- | --- | --- |
|  | **Haloperidol** | | **Quetiapine** | **Olanzapine** | **Risperidone** | **Ziprasidone** | **Aripiprazole** | | **Methotrimeprazine** |
|  | N= 36 | | N= 11 | N= 12 | N= 10 | N= 2 | N= 4 | | N= 1 |
| **Intensive care**  N= 24 | 24 (100%) | | 7 (29%) | 8 (33%) | 4 (17%) | 2 (10%) | 2 (8%) | | 0 (0%) |
| **Inpatient**  N= 8 | 8 (100%) | | 3 (38%) | 3 (38%) | 5 (63%) | 0 (0%) | 1 (13%) | | 1 (13%) |
| **Emergency department**  N= 4 | 4 (100%) | | 1 (25%) | 1 (25%) | 1 (25%) | 0 (0%) | 1 (25%) | | 0 (0%) |

Percentages do not add up to 100 due to the possibility of multiple outcomes per study

**Supplementary Table 7.** Number of studies reporting on measured outcomes of antipsychotic prescribing practices in the acute care setting, by acute care setting and antipsychotic type

| **ACUTE CARE SETTING** |  | **MEASURED ANTIPSYCHOTIC PRESCRIBING PRACTICES**  N= 34 | | | | | |  |  |
| --- | --- | --- | --- | --- | --- | --- | --- | --- | --- |
|  | **Haloperidol** | | **Quetiapine** | **Olanzapine** | **Risperidone** | **Ziprasidone** | **Aripiprazole** | | **Methotrimeprazine** |
|  | N= 26 | | N= 26 | N= 23 | N= 22 | N= 11 | N= 8 | | N= 0 |
| **Intensive care**  N= 20 | 14 (70%) | | 17 (85%) | 14 (70%) | 13 (65%) | 7 (35%) | 5 (25%) | | 0 (0%) |
| **Inpatient**  N= 14 | 12 (86%) | | 9 (64%) | 9 (64%) | 9 (64%) | 4 (29%) | 3 (21%) | | 0 (0%) |
| **Emergency department**  N= 0 | 0 (0%) | | 0 (0%) | 0 (0%) | 0 (0%) | 0 (0%) | 0 (0%) | | 0 (0%) |

Percentages do not add up to 100 due to the possibility of multiple outcomes per study

**Supplementary Table 8.** Reported additionally prescribed sedative hypnotic medications for included studies reporting on antipsychotic medication prescribing, by acute care setting

| REPORTED ADDITIONAL SEDATIVE HYPNOTIC MEDICATIONS | ACUTE CARE SETTING | | |
| --- | --- | --- | --- |
|  | **Intensive care**  N=28 | **Inpatient**  N=15 | **Emergency department**  N=1 |
| Benzodiazepines | 24 (86%) | 8 (53%) | 1 (0%) |
| Intravenous sedative infusions^1^ | 9 (32%) | 0 (0%) | 0 (0%) |
| Opioid pain medications | 8 (29%) | 2 (13%) | 0 (0%) |
| Dexmedetomidine | 8 (29%) | 1 (7%) | 0 (0%) |
| Another antipsychotic | 7 (25%) | 8 (53%) | 0 (0%) |
| Sleep aids | 2 (7%) | 1 (7%) | 0 (0%) |
| Other sedatives^2^ | 2 (7%) | 3 (20%) | 0 (0%) |
| Clonidine | 1 (4%) | 1 (7%) | 0 (0%) |

Percentages do not add up to 100 due to the possibility of multiple outcomes per study

^1^Reported propofol and ketamine infusions

^2^Reported barbiturates and hydroxyzine

**Supplementary Table 9.** Reported co-prescribed sedative hypnotic medications with antipsychotic medications for included studies which report on additionally prescribed medications, by acute care setting

| **Intensive Care** | | | | | | | | | | | |
| --- | --- | --- | --- | --- | --- | --- | --- | --- | --- | --- | --- |
| **First author** | **Year** | **Study type** | **Country/Continent** | **Reported additional sedative hypnotic medications** | | | | | | | |
|  |  |  |  | **Benzodiazepines** | **Intravenous sedative infusions^1^** | **Opioid pain medications** | **Dexmedetomidine** | **Another antipsychotic** | **Sleep aids** | **Other sedatives^2^** | **Clonidine** |
| Boncyk, C.S. | 2021 | Retrospective cohort study | United States |  |  |  |  | **✓** |  |  |  |
| Brown, G. | 1998 | Quasi-experimental study | Canada | **✓** |  |  |  | **✓** |  |  |  |
| Ceraso, D.H. | 2010 | Cross-sectional survey | South America^‡^ | **✓** |  |  | **✓** | **✓** |  |  |  |
| Collet, M.O. | 2019 | Focus groups | Denmark | **✓** |  |  |  |  |  |  |  |
| D’Angelo, R.G. | 2019 | Pre-post interventional study | United States | **✓** |  | **✓** |  |  |  |  |  |
| DeBacker, J. | 2018 | Retrospective cohort study | Canada | **✓** | **✓** | **✓** |  |  |  |  |  |
| Devlin, J.W. | 2011 | Cross-sectional survey | United States | **✓** |  |  | **✓** | **✓** |  |  |  |
| Dyal, S. | 2019 | Cross-sectional survey | United States | **✓** |  |  |  | **✓** |  | **✓** | **✓** |
| Dzierba, A.L. | 2019 | Cross-sectional survey | United States | **✓** | **✓** |  | **✓** |  |  |  |  |
| Ely, E.W. | 2004 | Cross-sectional survey | United States | **✓** |  |  |  |  |  |  |  |
| Farrokh, S. | 2017 | Retrospective cohort study | United States |  |  |  |  | **✓** |  |  |  |
| Gilani, A.A. | 2020 | Cross-sectional survey | United Kingdom |  |  |  |  | **✓** |  |  |  |
| Gilbert, B. | 2017 | Retrospective cohort study | United States | **✓** |  |  |  |  | **✓** |  |  |
| Gill, K.V. | 2012 | Cross-sectional survey + retrospective cohort study | United States | **✓** | **✓** |  |  |  |  |  |  |
| Glass, M. | 2018 | Pre-post interventional study | Not reported | **✓** |  |  |  |  |  |  |  |
| Levine, A.R. | 2019 | Retrospective cohort study | United States | **✓** |  | **✓** |  |  |  |  |  |
| Mac Sweeney, R. | 2010 | Cross-sectional survey | United Kingdom | **✓** | **✓** |  |  |  |  |  |  |
| Mehta, S. | 2007 | Cross-sectional survey | Canada | **✓** |  | **✓** |  |  |  |  |  |
| Mo, Y. | 2017 | Cross-sectional survey | United States | **✓** | **✓** |  | **✓** |  |  |  |  |
| Patel, M. | 2019 | Retrospective cohort study | Not reported |  |  |  |  |  | **✓** |  |  |
| Patel, R.P. | 2009 | Cross-sectional survey | United States | **✓** | **✓** | **✓** | **✓** |  |  |  |  |
| Ranzani, O.T. | 2014 | Quality improvement study | Brazil | **✓** | **✓** |  | **✓** |  |  |  |  |
| Rhoney, D.H. | 2003 | Cross-sectional survey | United States | **✓** | **✓** | **✓** |  |  |  |  |  |
| Salluh, J.I.F. | 2009 | Cross-sectional survey | Brazil | **✓** | **✓** |  | **✓** |  |  |  |  |
| Silverman, D. | 2013 | Retrospective cohort study | United States | **✓** |  |  |  |  |  |  |  |
| Sztrymf, B. | 2012 | Cross-sectional survey | France | **✓** |  |  |  |  |  | **✓** |  |
| Trogrlic, Z. | 2013 | Prospective cohort study | Netherlands | **✓** |  |  |  |  |  |  |  |
| Wang, J. | 2017 | Cross-sectional survey | China | **✓** |  |  | **✓** |  |  |  |  |
| **Inpatient** | | | | | | | | | | | |
| Al-Qadheeb, N.S. | 2013 | Prospective cohort study | United States | **✓** |  |  |  |  |  |  |  |
| Bascom, P.B. | 2014 | Case report or case series | USA | **✓** |  | **✓** |  |  |  | **✓** |  |
| Brennan, M. | 2018 | Case-control | USA |  |  |  |  | **✓** |  |  |  |
| Fontaine, G.V. | 2018 | Retrospective cohort study | United States |  |  |  |  | **✓** |  |  |  |
| Hosie, A. | 2021 | Cross-sectional survey | Australia (All) | **✓** |  |  | **✓** |  | **✓** | **✓** | **✓** |
| Hui, D. | 2011 | Retrospective cohort study | United States | **✓** |  |  |  |  |  | **✓** |  |
| Kuscu, M.K. | 2004 | Cross sectional survey + semi-structured interviews | Turkey | **✓** |  |  |  |  |  |  |  |
| Loh, E.C. | 2011 | Case report or case series | Malaysia |  |  |  |  | **✓** |  |  |  |
| Masman, A.D. | 2015 | Retrospective cohort study | Netherlands | **✓** |  | **✓** |  | **✓** |  |  |  |
| Meagher, D. | 2013 | Cross-sectional survey | Europe^†^ |  |  |  |  | **✓** |  |  |  |
| Thacker, S. | 1996 | Cross-sectional survey | United Kingdom | **✓** |  |  |  | **✓** |  |  |  |
| Trenaman, S.C. | 2018 | Cross-sectional cohort study | Canada |  |  |  |  | **✓** |  |  |  |
| Tropea, J. | 2009 | Medical record audit | Australia | **✓** |  |  |  | **✓** |  |  |  |
| **Emergency department** | | | | | | | | | | | |
| Chan, E.W. | 2011 | Cross-sectional survey | Australia | **✓** |  |  |  |  |  |  |  |

^1^Reported propofol and ketamine infusions

^2^Reported barbiturates and hydroxyzine

**Supplementary Table 10.** Domains and constructs according to the Theoretical Domains Framework of perspectives on antipsychotic prescribing from healthcare professionals for included studies, by acute care setting

|  | **Intensive care** | **Inpatient** | **Emergency department** |
| --- | --- | --- | --- |
| **Perspectives^1^** | **N=18** | **N=7** | **N=4** |
| **Knowledge**  Knowledge of  condition/scientific  rationale | 14 (77%)  14 (100%) | 6 (86%)  6 (100%) | 3 (75%)  3 (100%) |
| **Skills**  Skill development  Competence  Ability  Skill assessment  Interpersonal skills | 8 (44%)  3 (38%)  4 (50%)  1 (13%)  0 (0%)  0 (0%) | 2 (29%)  0 (0%)  1 (50%)  0 (0%)  0 (0%)  1 (50%) | 2 (50%)  0 (0%)  1 (50%)  0 (0%)  1 (50%)  0 (0%) |
| **Social/Professional Role and Identity**  Professional role  Professional confidence  Leadership | 9 (53%)  4 (44%)  4 (44%)  1 (11%) | 4 (50%)  2 (50%)  1 (25%)  1 (25%) | 3 (75%)    1 (33%)  2 (67%)  0 (0%) |
| **Beliefs about Capabilities**  Perceived competence  Self-efficacy  Perceived behavioral control  Beliefs  Empowerment | 16 (89%)  13 (81%)  2 (13%)  1 (6%)  1 (6%)  1 (6%) | 5 (71%)  3 (60%)  0 (0%)  1 (20%)  1 (20%)  0 (0%) | 4 (100%)  4 (100%)  0 (0%)  0 (0%)  0 (0%)  0 (0%) |
| **Optimism** | 0 (0%) | 1 (13%) | 0 (0%) |
| **Beliefs about Consequences**  Beliefs  Consequences  Outcome expectancies | 14 (78%)  6 (43%)  7 (50%)  2 (11%) | 6 (86%)  3 (50%)  4 (67%)  0 (0%) | 3 (75%)  0 (0%)  2 (67%)  1 (33%) |
| **Reinforcement**  Rewards  Reinforcement | 5 (29%)  2 (40%)  3 (60%) | 1 (13%)  1 (100%)  0 (0%) | 2 (50%)  1 (50%)  1 (50%) |
| **Intentions**  Stability of intentions  Stages of change model | 1 (6%)  0 (0%)  1 (100%) | 1 (13%)  0 (0%)  1 (100%) | 0 (0%)  0 (0%)  0 (0%) |
| **Goals**  Goal priority  Goal/target setting | 2 (12%)  2 (100%)  0 (0%) | 3 (38%)  2 (67%)  1 (33%) | 1 (25%)  0 (0%)  1 (100%) |
| **Memory, Attention & Decision Processes**  Decision making  Cognitive overload/tiredness  Attention | 7 (41%)  5 (71%)  1 (14%)  1 (14%) | 2 (25%)  2 (100%)  0 (0%)  0 (0%) | 1 (25%)    1 (100%)  0 (0%)  0 (0%) |
| **Environmental Context & Resources**  Environmental stressors  Resources/material  resources  Organizational  culture/climate  Person-environment  Interaction  Facilitators and barriers | 16 (94%)  1 (6%)  12 (75%)    1 (6%)  1 (6%)  1 (6%) | 2 (25%)  0 (0%)  1 (50%)  0 (0%)  0 (0%)  1 (50%) | 3 (75%)  0 (0%)  3 (100%)  0 (0%)  0 (0%)  0 (0%)  0 (0%) |
| **Social Influences**  Group norms  Group conformity  Social comparisons  Alienation  Intergroup conflict | 8 (47%)  3 (38%)  2 (25%)  1 (13%)  2 (25%)  1 (13%) | 2 (25%)  1 (50%)  0 (0%)  1 (50%)  0 (0%)  0 (0%) | 3 (75%)  2 (67%)  1 (33%)  0 (0%)  0 (0%)  0 (0%) |
| **Emotion**  Stress  Fear | 3 (18%)  2 (67%)  1 (33%) | 2 (25%)  1 (50%)  1 (50%) | 0 (0%)  0 (0%)  0 (0%) |
| **Behavioral Regulation**  Self-monitoring  Action planning | 4 (24%)  3 (75%)  1 (25%) | 3 (38%)  2 (67%)  1 (33%) | 1 (25%)  1 (100%)  0 (0%) |
|  |  |  |  |

Percentages do not add up to 100 due to the possibility of multiple outcomes per study

^1^Perspectives determined from deductive thematic analysis using the Theoretical Domains Framework of included studies

**Supplementary Table 11.** Deductive thematic analysis using the Theoretical Domains Framework on perceptions on antipsychotic prescribing for included studies

|  |  |  | **Domains and constructs^1^** | | | | | | | | | | | | | |
| --- | --- | --- | --- | --- | --- | --- | --- | --- | --- | --- | --- | --- | --- | --- | --- | --- |
| **First author** | **Year** | **Clinical specialty** | **Knowledge** | **Skills** | **Social/Professional Role** | **Beliefs about Capabilities** | **Optimism** | **Beliefs about Consequences** | **Reinforcement** | **Intentions** | **Goals** | **Memory, Attention & Decision Processes** | **Environmental Context & Resources** | **Social Influences** | **Emotion** | **Behavioral Regulation** |
| **Intensive care** | | | | | | | | | | | | | | | | |
| Almehairi, E. | 2018 | Intensive care | x | x | x | **✓** *(Perceived behavioral control)* | x | x | x | x | x | x | **✓** *(Resources/material resources)* | x | x | x |
| Ceraso, D.H. | 2010 | Intensive care | **✓** *(Knowledge)* | x | **✓** *(Professional role)* | **✓** *(Perceived competence)* | x | **✓** *(Consequences)* | x | x | x | x | **✓** *(Resources/material resources)* | x | x | x |
| Chawla, R. | 2013 | Intensive care | x | x | x | **✓** *(Perceived competence)* | x | **✓** *(Beliefs)* | x | x | x | x | **✓** *(Resources/material resources)* | x | x | x |
| Collet, M.O. | 2019 | Intensive care | x | **✓** *(Ability)* | **✓** *(Professional confidence)* | **✓** *(Perceived competence)* | x | **✓** *(Consequences)* | **✓** *(Rewards)* | x | **✓** *(Goal priority)* | **✓** *(Decision making)* | **✓** *(Resources/material resources)* | **✓** *(Social comparisons)* | **✓** *(Fear)* | **✓** *(Self-monitoring)* |
| Devlin, J.W. | 2011 | Intensive care | **✓** *(Knowledge)* | **✓** *(Competence)* | **✓** *(Professional role)* | **✓** *(Perceived competence)* | x | **✓** *(Beliefs)* | **✓** *(Rewards)* | x | x | **✓** *(Decision making)* | **✓** *(Resources/material resources)* | x | x | **✓** *(Action planning)* |
| Ely, E.W. | 2004 | Intensive care | **✓** *(Knowledge)* | x | **✓** *(Professional role)* | **✓** *(Perceived competence)* | x | **✓** *(Beliefs; consequences)* | **✓** *(Reinforcement)* | x | x | x | **✓** *(Resources/material resources)* | x | x | x |
| Flores, D.J. | 2015 | Intensive care | **✓** *(Knowledge)* | **✓** *(Skill development)* | x | **✓** *(Perceived competence)* | x | **✓** *(Outcome expectancies)* | x | x | x | x | **✓** *(Barriers and facilitators)* | **✓** *(Alienation)* | x | x |
| Gilani, A.A. | 2020 | Intensive care | x | x | x | x | x | x | x | x | x | **✓** *(Cognitive overload/tiredness)* | **✓** *(Resources/material resources)* | x | **✓** *(Stress)* | **✓** *(Self-monitoring)* |
| Gill, K.V. | 2012 | Intensive care | **✓** *(Knowledge)* | x | x | x | x | x | **✓** *(Reinforcement)* | **✓** *(Stages of change model)* | x | **✓** *(Decision making)* | **✓** *(Organizational culture/climate)* | **✓** *(Group norms)* | x | x |
| Johnson, K. | 2016 | Intensive care | **✓** *(Knowledge)* | **✓** *(Skill development)* | x | **✓** *(Perceived competence)* | x | **✓** *(Consequences)* | x | x | x | x | x | x | x | x |
| Mehta, S. | 2007 | Intensive care | **✓** *(Knowledge)* | **✓** *(Competence)* | **✓** *(Professional confidence)* | **✓** *(Self-efficacy)* | x | **✓** *(Beliefs)* | x | x | x | **✓** *(Decision making)* | **✓** *(Resources/material resources)* | **✓** *(Group conformity)* | x | x |
| Mo, Y. | 2017 | Intensive care | **✓** *(Knowledge)* | **✓** *(Skill assessment)* | x | **✓** *(Perceived competence)* | x | **✓** *(Beliefs)* | x | x | **✓** *(Goal priority)* | **✓** *(Decision making)* | **✓** *(Resources/material resources)* | x | x | x |
| Palacios-Ceña, D. | 2016 | Intensive care | **✓** *(Knowledge)* | **✓** *(Competence)* | **✓** *(Professional role)* | **✓** *(Perceived competence)* | x | **✓** *(Beliefs)* | x | x | x | **✓** *(Attention)* | **✓** *(Person-environment interaction)* | **✓** *(Group conformity; alienation)* | **✓** *(Stress)* | **✓** *(Self-monitoring)* |
| Patel, R.P. | 2009 | Intensive care | **✓** *(Knowledge)* | x | x | **✓** *(Perceived competence)* | x | **✓** *(Consequences)* | x | x | x | x | **✓** *(Resources/material resources)* | x | x | x |
| Ranzani, O.T. | 2014 | Intensive care | **✓** *(Knowledge)* | **✓** *(Competence)* | **✓** *(Leadership)* | **✓** *(Self-efficacy; perceived competence)* | x | x | x | x | x | x | **✓** *(Resources/material resources)* | **✓** *(Intergroup conflict)* | x | x |
| Sztrymf, B. | 2012 | Intensive care | **✓** *(Knowledge)* | x | **✓** *(Professional confidence)* | **✓** *(Perceived competence)* | x | **✓** *(Consequences)* | **✓** *(Reinforcement)* | x | x | x | **✓** *(Environmental stressors)* | **✓** *(Group norms)* | x | x |
| Trogrlic, Z. | 2013 | Intensive care | **✓** *(Knowledge)* | x | x | **✓** *(Empowerment; perceived competence)* | x | **✓** *(Outcome expectancies)* | x | x | x | x | x | x | x | x |
| Wang, J. | 2017 | Intensive care | **✓** *(Knowledge)* | x | **✓** *(Professional confidence)* | **✓** *(Beliefs)* | x | **✓** *(Consequences)* | x | x | x | x | **✓** *(Resources/material resources)* | **✓** *(Group norms)* | x | x |
| **Inpatient** | | | | | | | | | | | | | | | | |
| Bascom, P.B. | 2014 | Inpatient | **✓** *(Knowledge)* | **✓** *(Competence)* | **✓** *(Professional role)* | x | x | **✓** *(Consequences)* | x | x | **✓** *(Goal priority)* | x | x | x | **✓** *(Fear)* | x |
| Birigen, E.K. | 2021 | Inpatient | **✓** *(Knowledge)* | x | **✓** *(Professional confidence)* | **✓** *(Perceived competence)* | x | **✓** *(Consequences)* | x | x | x | **✓** *(Decision making)* | **✓** *(Resources/material resources)* | **✓** *(Group norms)* | x | **✓** *(Action planning)* |
| Hosie, A. | 2021 | Inpatient | **✓** *(Knowledge)* | **✓** *(Interpersonal skills)* | **✓** *(Leadership)* | **✓** *(Perceived competence)* | **✓** *(Optimism)* | **✓** *(Consequences)* | x | **✓** *(Stages of change model)* | **✓** *(Goal/target setting)* | **✓** *(Decision making)* | **✓** *(Resources/material resources)* | **✓** *(Social comparisons)* | **✓** *(Stress)* | x |
| Loh, E.C. | 2011 | Inpatient | x | x | **✓** *(Professional role)* | x | x | **✓** *(Beliefs)* | x | x | **✓** *(Goal priority)* | x | x | x | x | x |
| Meagher, D. | 2013 | Inpatient | **✓** *(Knowledge)* | x | x | **✓** *(Perceived competence)* | x | x | x | x | x | x | x | x | x | x |
| Someya, T. | 2001 | Inpatient | **✓** *(Knowledge)* | x | x | **✓** *(Beliefs)* | x | **✓** *(Beliefs)* | **✓** *(Rewards)* | x | x | x | x | x | x | **✓** *(Action planning)* |
| Yasuyuki, O. | 2016 | Inpatient | **✓** *(Knowledge)* | x | x | **✓** *(Perceived behavioral control)* | x | **✓** *(Beliefs)* | x | x | x | x | x | x | x | **✓** *(Self-monitoring)* |
| **Emergency department** | | | | | | | | | | | | | | | | |
| Bervoets, C. | 2015 | Emergency department | **✓** *(Knowledge)* | x | **✓** *(Professional role)* | **✓** *(Perceived competence)* | x | **✓** *(Consequences)* | x | x | x | x | x | **✓** *(Group norms)* | x | **✓** *(Self-monitoring)* |
| Chan, E.W. | 2015 | Emergency department | x | x | x | **✓** *(Perceived competence)* | x | x | x | x | x | x | **✓** *(Resources/material resources)* | **✓** *(Group conformity)* | x | x |
| Chan, E.W. | 2011 | Emergency department | **✓** *(Knowledge)* | **✓** *(Competence)* | **✓** *(Professional confidence)* | **✓** *(Perceived competence)* | x | **✓** *(Consequences)* | **✓** *(Reinforcement)* | x | x | x | **✓** *(Resources/material resources)* | **✓** *(Group norms)* | x | x |
| Cowling, M. | 2019 | Emergency department | **✓** *(Knowledge)* | **✓** *(Skill assessment)* | x | **✓** *(Perceived competence)* | x | x | **✓** *(Rewards)* | x | **✓** *(Goal/target setting)* | **✓** *(Decision making)* | x | x | x | x |

^1^Constructs reported in parentheses of appropriate domains

**Supplementary Table 12.** Description of reported antipsychotic deprescribing strategies applied in parallel for included studies reporting on antipsychotic medication prescribing

| First author | Year | Study Location | Clinical speciality | Deprescribing strategies |
| --- | --- | --- | --- | --- |
| D’Angelo, R.G. | 2019 | United States | Intensive care | 1. Antipsychotic discontinuation algorithm  implemented before ICU transfer  2. Multidisciplinary education of algorithm |
| Kram, B.L. | 2019 | United States | Intensive care | 1. Pharmacy-based electronic handoff tool  2. Pharmacist education on ICU delirium and  consensus guidelines for antipsychotic use |
| Stuart, M.M. | 2020 | United States | Intensive care | 1. Pharmacist-driven prescriptive authority to  discontinue or taper antipsychotic medication  following resolution of delirium  2. Collaborative practice agreement between  physicians and pharmacists |
